# Thymocyte regulatory variant alters transcription factor binding and protects from type 1 diabetes in infants

**DOI:** 10.1038/s41598-022-18296-4

**Published:** 2022-08-19

**Authors:** Niina Sandholm, Arcadio Rubio García, Marcin L. Pekalski, Jamie R. J. Inshaw, Antony J. Cutler, John A. Todd

**Affiliations:** 1 JDRF/Wellcome Diabetes and Inflammation Laboratory, Wellcome Centre for Human Genetics, Nuffield Department of Medicine, NIHR Oxford Biomedical Research Centre, University of Oxford, Oxford, UK; 2 Folkhälsan Research Center, Helsinki, Finland; 3 Research Program for Clinical and Molecular Metabolism, Faculty of Medicine, University of Helsinki, Helsinki, Finland; 4 Department of Paediatrics, University of Oxford, Oxford, UK

**Keywords:** Data integration, Type 1 diabetes, Genetics research, Computational biology and bioinformatics, Epigenetics, Epigenomics, Gene regulation, Genetics

## Abstract

We recently mapped a genetic susceptibility locus on chromosome 6q22.33 for type 1 diabetes (T1D) diagnosed below the age of 7 years between the *PTPRK* and thymocyte-selection-associated (*THEMIS)* genes. As the thymus plays a central role in shaping the T cell repertoire, we aimed to identify the most likely causal genetic factors behind this association using thymocyte genomic data. In four thymocyte populations, we identified 253 DNA sequence motifs underlying histone modifications. The G insertion allele of rs138300818, associated with protection from diabetes, created thymocyte motifs for multiple histone modifications and thymocyte types. In a parallel approach to identifying variants that alter transcription factor binding motifs, the same variant disrupted a predicted motif for Rfx7, which is abundantly expressed in the thymus. Chromatin state and RNA sequencing data suggested strong transcription overlapping rs138300818 in fetal thymus, while expression quantitative trait locus and chromatin conformation data associate the insertion with lower *THEMIS* expression. Extending the analysis to other T1D loci further highlighted rs66733041 affecting the GATA3 transcription factor binding in the *AFF3* locus. Taken together, our results support a role for thymic *THEMIS* gene expression and the rs138300818 variant in promoting the development of early-onset T1D.

## Introduction

Over 1 million children and adolescents have type 1 diabetes (T1D), a disease caused by an autoimmune reaction against the insulin-producing beta cells in the pancreatic islets. The HLA class II and I genes play an important role in the development of T1D and other autoimmune diseases, with the strongest risk conveyed by the HLA class II DRB1*03-DQB1*02/DRB1*0401-DQB1*0302 genotype. In addition, over 100 other genetic loci have been associated with T1D^1–4^.

The HLA proteins bind pathogen and self-antigen fragments, peptides, which are then recognized by highly variable T cell antigen receptors (TCR), found on the T cell surface. In the thymus, the T cell precursors, called thymocytes, are first matured from CD4^-^CD8^-^ double-negative cells to CD4^+^CD8^+^ double-positive cells. In positive selection, thymocytes are limited to those with sufficient TCR binding to the HLA proteins. The cells with affinity to HLA class II proteins found on antigen-presenting cells differentiate towards CD4^+^ T-helper cells; and the ones with affinity to HLA class I proteins expressed by most cells differentiate towards CD8^+^ T-killer cells. Negative selection then excludes the T cells targeting the self, thus protecting from autoimmunity^5^. Therefore, thymus has been suggested to play a role in the development of T1D and other autoimmune diseases^6,7^. Support for this hypothesis came from a genome-wide association study (GWAS) on T1D where the susceptibility loci were enriched on active enhancer sites in the thymus, in addition to T, B, and NK cells and CD34^+^ stem cells^8^. Furthermore, a recent single-cell RNA sequencing (scRNA-seq) study on developing mouse thymus identified a modest enrichment of GWAS signals from T1D and other autoimmune diseases especially in the thymus blood cell populations^9^.

The training of the immune system occurs particularly in early childhood when the body is adapting to the changing environment. The thymus itself is proportionally largest at birth, and nearly disappears in adults. Therefore, the role of the thymus in autoimmunity is likely most pronounced for childhood autoimmune diseases. For example, we have discovered in the infant gut microbiome a cross-reactive mimotope of insulin—the primary autoantigen in T1D—encoded in the commensal bacterial transketolase enzyme^10^. Recently it has been shown in mice that intestinal dendritic cells can engulf gut bacteria and traffic to the thymus where the bacterial antigens are presented to the T cell immune system^11^. Hence, expression of insulin in the human thymus is protective for T1D and now the expression of bacterial antigens early in life in the thymus adds to the body’s attempts to establish and maintain immune tolerance to insulin.

Several of the T1D-associated HLA and non-HLA variants lower the age-at-diagnosis (AAD) of T1D^12^. Our recent genetic analysis identified a novel locus on chromosome 6q22.33 associated with earlier diagnosis of T1D (rs72975913, *p* = 2.94 × 10 ^− 10^)^13^; the locus was associated with diabetes particularly among those diagnosed in the first 7 years^12^. The association is located between the thymocyte selection associated (*THEMIS*) and Protein tyrosine phosphatase, receptor type K (*PTPRK*) genes. The locus has been previously associated with other autoimmune diseases including multiple sclerosis^14^ and Crohn’s disease^15^. Colocalization analysis of the expression quantitative trait locus (eQTL) data in whole blood suggested that the AAD association co-localises with the *THEMIS* eQTL signal such that the minor alleles—protective for T1D—were associated with decreased *THEMIS* expression; and to limited extend with the *PTPRK* expression^12^. *THEMIS* plays a regulatory role in both positive and negative T cell selection during late thymocyte development^16^. Specifically in the positive selection, *THEMIS* inactivates the tyrosine phosphatase SHP-1 and thereby lowers the TCR signalling threshold, enabling thymocytes to be stimulated by weak agonists^17^. While *PTPRK* is widely expressed and regulates a variety of cellular processes including cell growth and differentiation, studies of the spontaneous deletion in Long Evans Cinnamon (LEC) rats indicated that also *PTPRK* is required for the thymocyte maturation into CD4^+^ positive T cells, and that the *PTPRK* expression in the thymus peaks in the double-negative thymocytes^18^.

**Figure 1:**
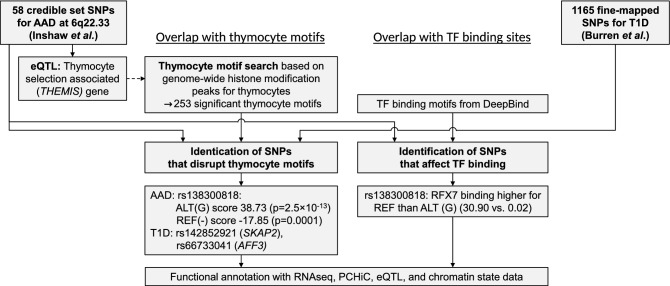
Two parallel approaches were utilized to identify age-at-diagnosis (AAD) and type 1 diabetes (T1D) credible set SNPs that affect thymocyte regulatory motifs or transcription factor (TF) binding motifs. Genetic variants on chromosome 6q22.33 locus near the thymocyte selection associated gene, *THEMIS,* are associated with the AAD and a lower risk of early-onset type 1 diabetes. The 58 credible set SNPs on this locus were obtained from the original analysis by Inshaw et al*.*^13^. *THEMIS* is responsible for the thymocyte selection in the thymus, a process thought to affect the development of T cell-related autoimmune diseases such as T1D. We sought DNA motifs located in active chromosome regions of thymocyte cells, defined as histone modification peaks and overlapping RNA expression, indicative of active enhancer regions^25^. Resulting thymocyte motifs were integrated with the 58 credible set variants on the 6q22.33 locus, and 1165 other SNPs for T1D, to identify SNPs that disrupt DNA motifs identified in the thymocyte epigenetic data. As a parallel approach, we applied a deep-learning method to identify the credible set variants that alter TF binding motifs.

Variants associated with common diseases are enriched in transcription factor (TF) binding sites and active chromatin regions, suggesting a regulatory role for disease outcome^19^. Indeed, it has been shown that DNA sequence variants can disrupt TF binding, leading to altered gene expression^20^. In this study, we hypothesized that the association at the recently identified 6q22.33 locus near the *THEMIS* gene is mediated through regulatory processes in the thymocytes. Utilizing extensive genomic data from the thymus and other tissues, combined with deep-learning-base prediction of TF binding sites, we aimed to pinpoint the likely causal variants and the functional mechanisms underlying the association (Fig. 1).

## Results

Statistical fine mapping of the 6q22.33 locus previously suggested 58 variants (single nucleotide polymorphisms or insertion-deletion [indel] variants, jointly abbreviated here as “SNPs”) in the credible set of likely causal variants across three linkage disequilibrium (LD) blocs; the posterior probability of the underlying causal variant being within the three blocks was estimated 0.96, 0.50, and 0.42, respectively^13^. Look-up of the variants in the promoter capture Hi-C (PCHiC) data for chromatin interactions indicated that many of the 58 SNPs were located in DNA fragments that interact with the *PTPRK* (in foetal thymus, naïve CD4^+^ T cells) and *THEMIS* (in Naïve CD8^+^ T cells) promoter regions (ChiCAGO score ≥ 5^21^; Supplementary Table S1 online). The SNPs interacted with no other genes in the thymus or in the 16 primary hematopoietic cells, and as a negative control, they had no PCHiC interactions in the pancreatic islets^22^. The strongest gene expression of *THEMIS* is found in the thymus in the FANTOM5 database^23^, and it is also expressed in T cells; also *PTPRK* had detectable expression in the thymus. In a recent scRNA-seq of developing human immune system including 108,615 thymus cells^24^, *THEMIS* was expressed particularly in the CD4^+^, CD8^+^, double negative and double positive T cells, as well as cycling T cells and type 1 and 3 innate T cells; *PTPRK* expression was highest in the same cell types (Supplementary Fig. S1).

### DNA sequence motifs in thymocytes

With the hypothesis that the associated SNPs affect the gene regulation in thymocytes, we set to search for regulatory DNA motifs in thymocytes, affected by the credible set of SNPs (Fig. 1). Thymocyte regulatory regions were defined as histone modification peaks and overlapping RNA expression, indicative of active enhancer regions^25^. Genome-wide histone modification marks, including H3K4me3, H3K4me1, H3K27ac, H3K36me3, and H3K27me3, were available from the Blueprint epigenomics project^26^ for thymocytes that were dissected to four cell types (CD3 ^− ^CD4^+^CD8^+^, CD3^+^CD4^+^CD8^+^, CD4^+^αβ, and CD8^+^αβ). The number of peaks ranged from 7088 to 55,622 per experiment (Supplementary Table S2).

DNA sequence motifs reoccurring within these histone modification peaks were sought with the MEME software MEME-ChIP tool^27^, yielding 150 partially overlapping motifs (E-value < 2.94 × 10 ^− 5^, Table 1). In addition, we obtained 103 motifs for chromatin pseudostates^28^ that consisted of combinations of histone modification marks pooled over the individuals (Supplementary Table S3). Among these total of 253 motifs, 48 motifs were similar (q-value < 0.01) to known binding motifs for SP1, SP2, SP3, SP4, KLF5, KLF16, ZNF263, mouse Zfp281 (human ortholog ZNF281), mouse Zfp740 (human ortholog ZNF740), Zfx, and RREB1 (Supplementary Table S4, Supplementary Fig. S2). While most of these TFs are expressed in nearly all tissues, it is of note that SP3 has the highest expression in thymus across the FANTOM5 tissues (159.9 transcripts per million [tpm]), along with high thymus expression of ZFP281 (43.2 tpm) as well.

**Table 1:** Number of histone modification peaks in genome, and identified motifs per cell type.

Histone mod	Cell type	N samples	N peaks	N motifs
H3K4me3	CD3^+^CD4^+^CD8^+^	3	48,295	17
	CD3^-^CD4^+^CD8^+^	2	33,132	19
	CD4^+^αß	1	37,452	20
	CD8^+^αß	1	36,825	18
H3K4me1	CD3^+^CD4^+^CD8^+^	1	36,308	2
H3K27ac	CD3^+^CD4^+^CD8^+^	2	33,131	18
	CD3^-^CD4^+^CD8^+^	1	12,334	17
	CD4^+^αß	1	6011	18
H3K36me3	CD3^+^CD4^+^CD8^+^	2	53,760	1
H3K27me3	CD3^-^CD4^+^CD8^+^	1	2333	20

N samples: number of samples available for the cell type and histone modification. N peaks: number of peaks after data preprocessing and pooling all peaks from the same cell type. N motifs: Number of significant DNA motifs (E-value < 2.94 × 10 ^− 5^) underlying cell-type-specific histone modification peak data, identified with MEME software.

### Credible set SNPs affecting thymocyte motifs

**Figure 2:**
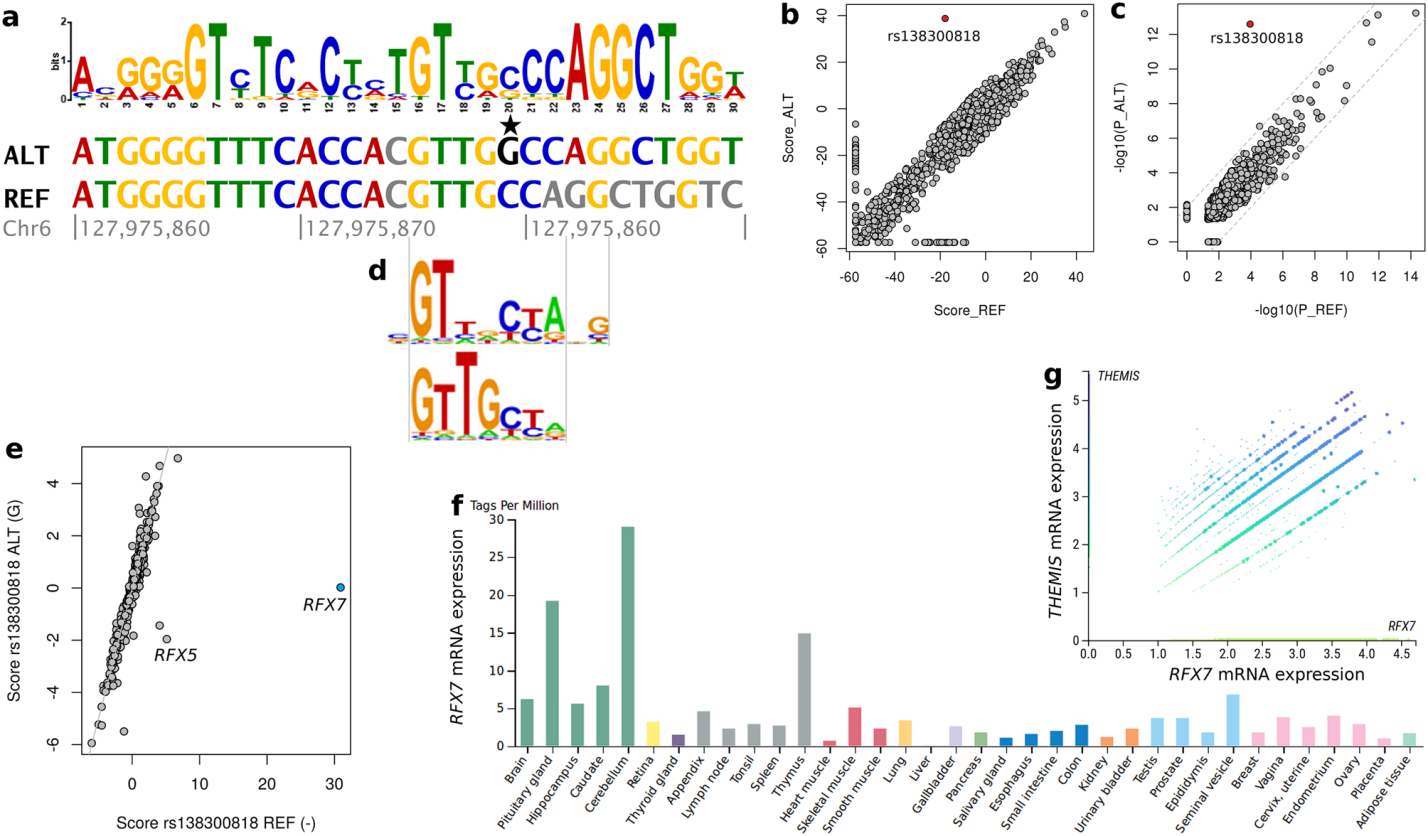
rs138300818 G insertion allele significantly increases sequence similarity with a thymocyte H3K27ac motif in CD4^+^αß cells. (**a**) Overlap between a thymocyte H3K27ac motif and rs138300818 alternative G allele (highlighted with star) and reference null allele flanking sequence. Bases conflicting with the motif are indicated with gray color. Panels (**b**) and (**c**)**:** Sequence similarity score (**b**) and −log_10_(*p*-value) distributions (**c**) for all credible set SNP—motif pairs where at least one of the SNP alleles (REF or ALT) forms a motif overlapping sequence (*p* < 0.05). rs138300818 is indicated as a red dot. (**d**) rs138300818 REF allele, but not ALT insertion allele, forms a RFX7 transcription factor binding site. The motifs are aligned with panel (**a**) sequence. (**e**) Score distribution for rs138300818 REF and ALT alleles against all available transcription factor binding sites from DeepBind database. (**f**) *RFX7* gene expression is highest in brain (cerebellum, pituitary gland), and in thymus in FANTOM5 database. (**g**) *RFX7* and *THEMIS* mRNA expression in early double negative [DN], DN (P/Q), double positive [DP] (P/Q), αβ entry, CD4^+^ , CD8^+^ , CD8AA, Treg, type 1/3 innate, and cycling T cells in scRNAseq of thymus. The DN (Q), DP (Q), and αβ entry T cells with the highest *RFX7* expression are highlighted with larger point size.

We then evaluated whether the 253 obtained sequence motifs for the thymocyte regulatory regions match the DNA sequences flanking the 58 credible set SNPs on the chr6q22.33 locus (Fig. 1). Indeed, many of the motifs overlapped the 58 AAD SNPs. For five SNPs, the flanking DNA sequences were similar to thymocyte motifs (*p* < 5.07 × 10 ^– 7^ for REF or ALT) and had significantly different *p*-value for the REF and ALT alleles (E_diff_ (P_REF _− P_ALT_) < 5.88 × 10 ^– 4^; Supplementary Table S5). The most marked allelic difference was observed for the rs138300818 insertion variant for H3K27ac in the CD4^+^αß cells, with the score for motif-sequence similarity of 38.73 (*p* = 2.5 × 10 ^– 13^) for the alternative G-insertion allele, and − 17.85 (*p* = 0.0001) for the reference allele (Fig. 2A–C). This was the only variant with a significant allelic difference in the score (E_diff_(Sc_REF _− Sc_ALT_) < 5.88 × 10 ^– 4^). A similar motif affected by the same rs138300818 substitution was identified in altogether 16 cell type and histone modification combinations, including all the four studied thymocyte cell types, and H3K27ac, H3K4me3 and H3K27me3 peaks. These thymocyte regulatory motifs overlapping rs138300818 did not resemble any known vertebrate TF-binding motifs evaluated with the tomtom sequence similarity analysis (E > 0.05).

### Credible set SNPs affecting TF binding motifs

As regulatory GWAS variants have been suggested to exert their effect specifically through affecting TF binding motifs in DNA^20^, we utilized DeepBind,a deep learning-based method that predicts the affinity of TFs to DNA sequences^29^, as a parallel approach to identify the credible set SNPs that alter TF binding motifs (Fig. 1). Among all the 58 SNP × 927 TF combinations, the strongest evidence of TF binding was identified for mouse Rfx7 binding to the rs138300818 REF allele flanking sequence; furthermore, the score for Rfx7 binding was markedly higher for the REF null allele than for the ALT (G) allele (30.90 vs. 0.02, respectively; score mean 0.07, sd 1.39 for all transcripts; Fig. 2D–F). Of note, this represented the largest allelic difference in predicted TF binding across all credible set SNPs for AAD and available TFs (Supplementary Fig. S3). Binding affinity was predicted higher for the reference allele also for two other RFX TF family members, human RFX5 and RFX3 (Supplementary Table S6). In developing human thymic cells, *RFX7* gene expression correlated with *THEMIS* expression especially in the double negative (Q), double positive (Q), and αβ entry T cells (Fig. 2G**, **Supplementary Figure S4).

We further utilized the sTRAP tool as an additional approach to calculate the affinity of TFs to the rs138300818 flanking sequence; the method is based on a biophysical model of the binding energies between the transcription factors and DNA^30^. Among the 1241 studied JASPAR transcription factors, the strongest difference between the rs138300818 REF and ALT alleles was obtained for Rfx4 (P_REF_ = 0.0025, p_ALT_ = 0.52, difference in log_10_ (*p*) = 2.32), Rfxdc2, and Rfx3, supporting the DeepBind findings (Supplementary Table S7). Of note, Rfx7 and Rfx5 motifs were not available for the analysis. Among all the 58 × 1241 SNP–TF combinations, the difference between Rfx4 binding affinity for rs13800818 REF vs ALT allele ranked within the top 0.003% (Supplementary Figure S5).

### Functional annotation for rs138300818

**Figure 3:**
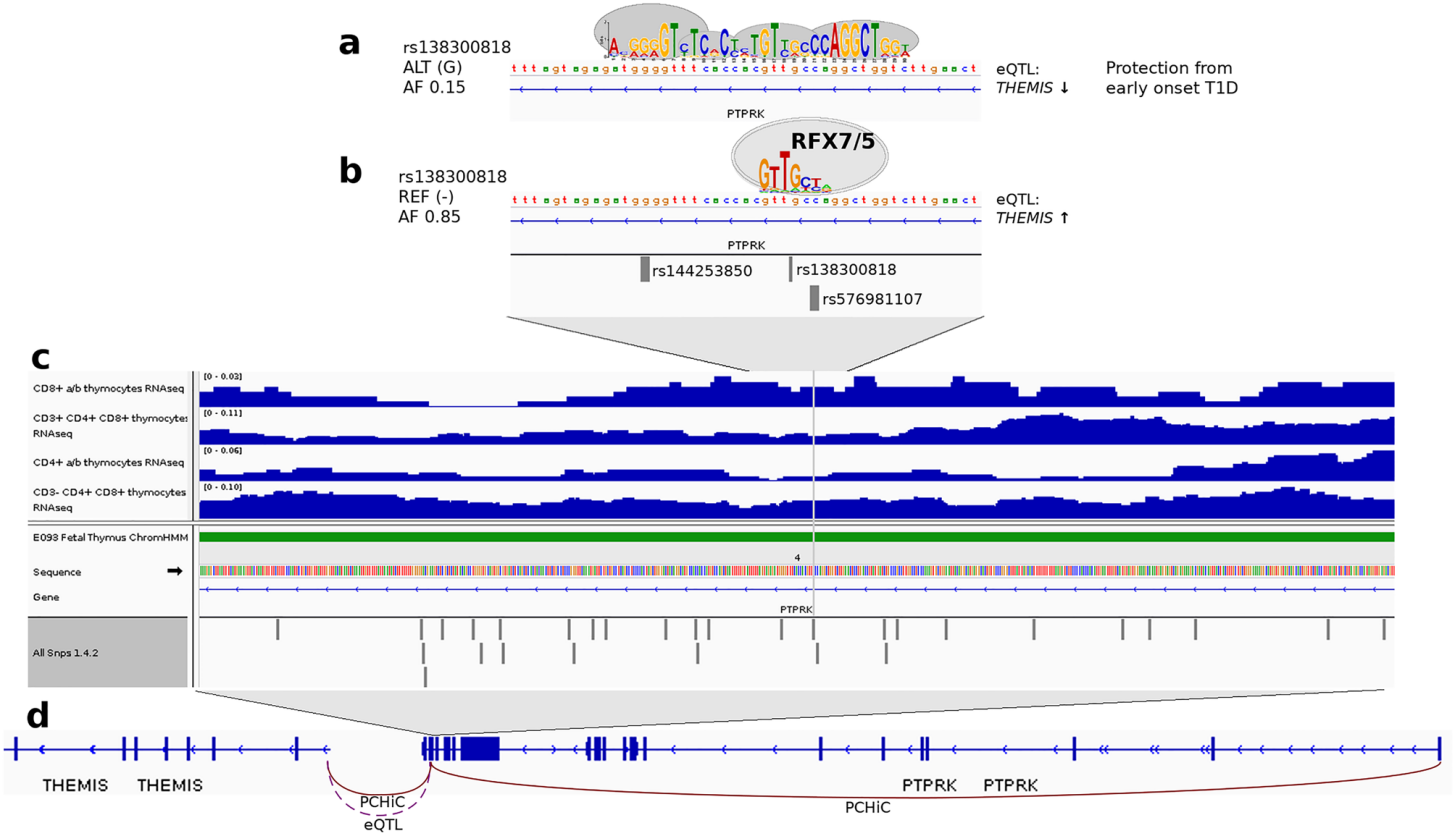
rs138300818 G insertion allele—protecting from early-onset T1D—creates a thymocyte motif (**a**). At the same time, this motif disrupts the RFX5/7 binding motif which is present with the rs138300818 reference (null) allele (**b**). rs138300818 is located intronic in *PTPRK*. Non-coding RNA transcription was detected overlapping rs138300818 (grey vertical line) in all four studied thymocyte cell types (dark blue peaks). Roadmap Epigenomics chromatin state data for fetal thymus (PrimaryHMM) indicated strong transcription (state 4, green bar) overlapping the SNP flanking region (**c**). PCHiC data in fetal thymocytes and in naïve CD8 cells suggests that rs138300818 interacts with both *PTPRK* and *THEMIS* transcription start sites. The genetic association signal for early-onset T1D is colocalized with eQTL signal for *THEMIS* in human whole blood; rs138300818 reference allele—which forms the RFX7/5 binding motif—is associated with higher *THEMIS* expression (**d**).

The variant rs138300818 has a minor allele frequency (MAF) of 0.155 (1000 Genomes [1000G] European population) for the G allele insertion, and it belongs to the highest 96% posterior probability group for association with AAD on the 6q22.33 locus^13^. The variant is an intronic variant within the *PTPRK* gene. Roadmap Epigenomics fetal thymus ChromHMM model^28^—based on histone modification data—predicted strong transcription overlapping rs138300818 (Fig. 3). Indeed, non-coding RNA expression—indicative of an active regulatory region^25^—was detected overlapping rs138300818 in all four studied thymocyte cell types (Fig. 3). PCHiC data for rs138300818 indicated interaction with both *PTPRK* (CHiCAGO scores 13.79 and 1.81, in the thymus and naïve CD8 cells, respectively), and with *THEMIS* (CHiCAGO scores 2.25 and 5.65, in the thymus and naïve CD8 cells, respectively).

### T1D credible set SNPs in other loci affecting thymocyte motifs

**Figure 4:**
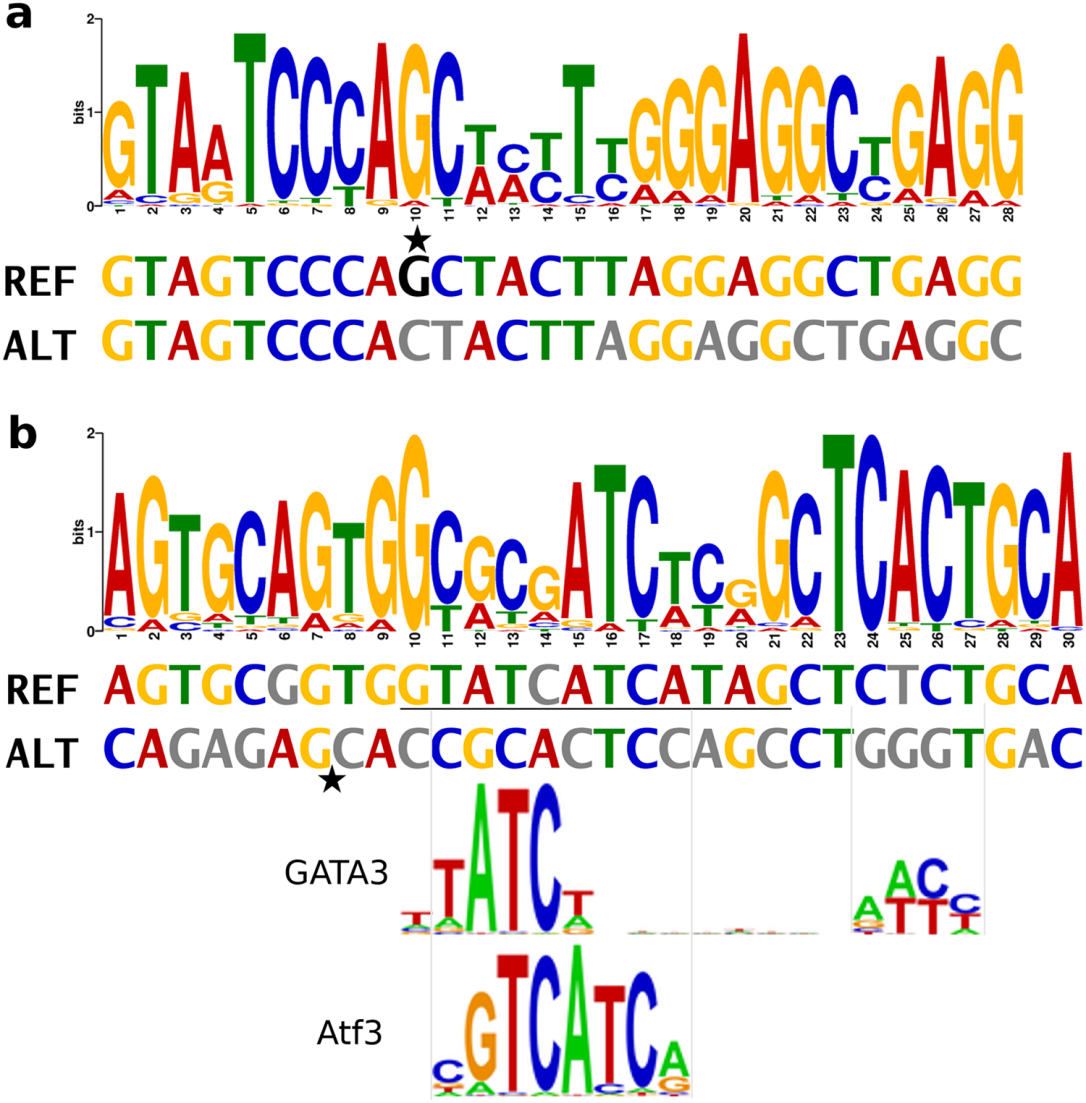
Two T1D susceptibility SNPs have allele-specific thymocyte motifs. (**a**) The reference G insertion allele of rs142852921 on chromosome 7p15.2 (*SKAP2*) region matches a state 10 motif (distal active promoters) in CD3^+^CD4^+^CD8^+^ cells (REF *p* = 4.5 × 10 ^− 15^, score 43.5; ALT *p* = 5.0 × 10 ^− 5^, score −11.6). (**b**) In the *AFF3* locus, rs66733041 reference allele (underlined sequence) forms a H3K4me3 motif in CD4^+^αβ cells (*p* = 1.4 × 10 ^− 11^, score 31.1), as well as GATA3 and Aft3 binding motifs, all of which are disrupted by the 12 bp deletion allele (indicated with a star). For better match, the ALT sequence was converted to reverse complement format.

We extended the search of overlapping thymocyte motifs to other T1D susceptibility loci. Out of 1,165 examined credible set variants from 20 susceptibility loci^31^, 9% demonstrated allele-specific sequence similarity to thymocyte motifs (E_diff_ (P_REF _− P_ALT_) < 5.88 × 10^ − 4^); however, only two variants had significant allelic difference in scores (E_diff_ (Sc_REF _− Sc_ALT_) < 5.88 × 10^ − 4^; Table 2). The largest difference was observed for rs142852921 on chromosome 7p15.2, with the reference G insertion allele matching a pseudostate 10 motif (distal active promoters) in CD3^+^CD4^+^CD8^+^ cells (*p* = 4.49 × 10^ − 15^ vs. *p* = 5.04 × 10^ − 5^ for alternative allele; Fig. 4A). Similar allele-specific motifs were observed for rs142852921 for altogether 12 thymocyte cell type—histone modification combinations. rs142852921 belongs to the fine-mapping block with 0.95 posterior probability, with the reference G insertion associated with higher T1D risk^13^. We detected non-coding RNA expression in thymocyte cells overlapping rs142852921 (Supplementary Fig. S4). rs142852921 G insertion is associated with lower *SKAP2* expression in whole blood (*p* = 3.5 × 10^ − 13^, m-value = 1) and other tissues, but also with higher *SKAP2* expression in brain (cerebellum *p* = 3.5 × 10^ − 10^, m-value = 0) and lower expression of homeobox genes (e.g., *HOXA7* whole blood *p*-value < 4.8 × 10^ − 7^).

**Table 2:** Thymocyte motifs overlapping credible set SNPs for AAD and T1D with marked difference in motif-sequence similarity between the reference and alternative alleles (E-value for difference in scores < 5.88 × 10 ^− 4^). For each SNP, only the histone modification and cell type combination with the largest allelic difference is shown.

Pheno	SNP	REF/ALT	Locus (Gene)	P_REF_	P_ALT_	Sc_REF_	Sc_ALT_	E_diff_(*P*)	E_diff_(Sc)	HistMod	Cell type
AAD	rs138300818	-/G	6q22.33 (*THEMIS-PTPRK*)	1.12 × 10 ^− 4^	2.53 × 10 ^− 13^	−17.9	38.7	2.51 × 10 ^− 29^	6.57 × 10 ^− 7^	H3K27ac	CD4^+^αβ
T1D	rs142852921	G/-	7p15.2 (*SKAP2*)	4.49 × 10 ^− 15^	5.04 × 10 ^− 5^	43.5	﻿−11.6	5.4 × 10 ^− 50^	3.6 × 10 ^− 7^	Pseudostate 10	CD3^+^CD4^+^CD8^+^
T1D	rs66733041	CT(ATG)_2_ATAC/-	2q11.2 (*AFF3)*	1.35 × 10 ^− 11^	0.0067	31.3	−41.2	3.0 × 10 ^− 32^	3.8 × 10 ^− 4^	H3K4me3	CD4^+^αβ

AAD, age-at-diabetes onset; T1D, type 1 diabetes. P_REF_ and P_ALT_: *P*-value for similarity between the thymocyte motif and the SNP flanking sequence with the REF and ALT alleles, respectively. Sc_REF_ and Sc_ALT_: Score for similarity between the thymocyte motif and the SNP flanking sequence with the REF and ALT alleles, respectively. E_diff_(P): E_diff_ (P_REF _− P_ALT_), i.e., E-value for the difference between the P_REF_ and P_ALT_. E_diff_(Sc): E_diff_ (Sc_REF _− Sc_ALT_) i.e., E-value for the difference between the Sc_REF_ and Sc_ALT_. HistMod: Histone modification. Pseudostate 10 corresponds to H3K4me3, H3K27Ac, and H3K4me1, “distal active promoters”.

Second, the reference allele of rs66733041 on *AFF3* locus matched a thymocyte motif (Fig. 4B, Supplementary Table S7) and binding sites for Atf3 and GATA3 TFs (DeepBind, Supplementary Table S6), which were all disrupted by the 12 bp deletion allele. The reference allele was associated with lower *AFF3* expression in brain and artery tissue (aorta, *p*-value = 2.9 × 10^ − 10^, m-value = 1). Neither of these *SKAP2* or *AFF3* variants had PCHiC interactions in thymocytes or in primary blood cells.

## Discussion

Recent genetic analyses have reported a novel susceptibility locus associated with early diagnosis of T1D between the *PTPRK* and *THEMIS* genes^13,32^. As the thymus is likely to play a major role in early-onset autoimmune diseases, and the *THEMIS* gene contributes to the thymocyte selection in the thymus, we hypothesised that the underlying causal genetic variant would affect a regulatory motif active in thymocytes. Based on histone modification peak calls across the genome in four thymocyte cell types, we identified DNA sequence motifs recurring in these regulatory regions in thymocytes (Fig. 1). One of the SNPs associated with early-onset T1D in the *PTPRK-THEMIS* locus significantly affected the identified thymocyte motifs: the minor G insertion allele of rs138300818, protecting from early-onset T1D, created a thymocyte motif in multiple thymocyte cell populations.

A parallel, independent approach to identify among the 58 credible set SNPs those that alter TF binding motifs—based on a deep learning method of experimental data—indicated that the same rs138300818 G insertion allele disrupted a TF binding motif for Rfx7 and RFX5, two structurally similar RFX family members (Fig. 2). Furthermore, another model to calculate the affinity of TFs to DNA sequences based on biophysical models suggested that the rs138300818 reference null allele had markedly higher binding affinity for Rfx4, Rfxdc2, and Rfx3. RFX7 is abundantly expressed in the thymus and lymphoid organs^33^. Rfx7 coordinates a transcriptional network controlling cell metabolism in natural killer (NK) cells, and Rfx7 binding sites are found in promoter regions of genes either up or down-regulated upon *Rfx7* knock-out in mice^33^. RFX5 regulates transcription of HLA class II genes, and mutations inactivating *RFX5* cause bare lymphocyte syndrome, a severe condition with primary HLA class II deficiency^34^. In the developing human thymus^24^, *RFX7* was coexpressed with *THEMIS* in the same T cell populations (Supplementary Figure S4).

The regulatory relevance of rs138300818 was further strengthened by the detection of non-coding RNA expression in all the studied thymocyte cells overlapping rs138300818. eQTL data suggested that the RFX5/7 binding rs138300818 reference allele is associated with higher *THEMIS* expression on whole blood^12^. Of note, functional studies of multiple sclerosis lead SNP rs13204742, in moderate LD with rs138300818 (*D'* = 0.91, *r*^2^ = 0.68), found that the major G allele was associated with higher *THEMIS* gene and exon 1 expression in CD4^+^ and CD8^+^ T cells *ex vivo*^35^. Through LD, this finding extends also to rs138300818. Finally, the role of the rs138300818 variant in early-onset T1D was further supported by chromatin conformation interactions with both *PTPRK* and *THEMIS* in the thymus and naïve CD8 cells.

*THEMIS* contributes to both positive^17^ and negative^16^ thymocyte selection, whereby dysregulation of the negative selection is believed to contribute to T-cell-mediated autoimmune diseases such as T1D^36^. In the rat, a spontaneous frameshift mutation in Themis leading to its disruption, resulted in a reduction of CD4^+^ cells in the thymus, further leading to reduction of blood CD4^+^ T lymphocytes; the mutation was associated with the development of inflammatory bowel disease^37^. Whereas the role of *THEMIS* is evident in thymocytes, also *PTPRK* has been implicated in regulating the development of CD4^+^ T cells^18^ in addition to a variety of other cellular processes. Of note, *PTPRK* is contiguous to *THEMIS* in all sequenced vertebrates, and the three *Themis*-like genes in vertebrate genomes are located close to *Ptprk* paralogs^38^. Altogether the results suggest that the rs138300818 insertion creates a common regulatory thymocyte motif that simultaneously disrupts the RFX5/7 motif, interfering with the *THEMIS* (and *PTPRK*) gene expression, leading to protection from early-onset T1D.

We further extended the analysis to all previously fine-mapped T1D SNPs. Two out of the 1,165 studied T1D-associated variants significantly affected a thymocyte motif. In the *AFF3* locus, a 12 basepair insertion allele at rs66733041 created a thymocyte motif and TF binding motifs for Atf3 and GATA3. GATA3 protein expression is upregulated in the early double-negative thymocytes^39^ and the protein plays an important role throughout the T cell development^40^. GATA3 directly regulates many critical genes and TFs both in the thymus and in peripheral T cell lineages, while demonstrating cell-type-specific binding patterns and gene regulation^41^. Furthermore, GATA3 was found to regulate active and repressive histone modifications at target enhancers, supporting the relevance of our approach^41^. Interestingly, variants in the *GATA3* gene were recently identified as a novel susceptibility locus for T1D^3^.

All the three variants with a significant allelic difference for thymocyte motif similarity (E_diff_ (Sc_REF _− Sc_ALT_) < 5.88 × 10^ − 4^) were indel variants. Indeed, indels have been reported to have an important role in modifying TF binding sites, and thus, regulating gene expression^42^. When considering only the less stringent allelic difference in *p*-values (E_diff_ (P_REF_ − P_ALT_) < 5.88 × 10^ − 4^), many more variants demonstrated allelic difference. As many disease-associated non-indel variants have been suggested to alter TF binding sites^29,43^, the comparative score distribution employed in this work may be too stringent and less sensitive to non-indel variants and capture only the most striking differences.

The main limitation of this work is the lack of functional work to confirm the findings. Nevertheless, it is of note that both our parallel, independent approaches to search for SNPs altering either thymocyte or TF binding motifs point to the same rs138300818 variant. Furthermore, existing functional work shows that a variant in LD with rs138300818 is associated with *THEMIS* expression in CD4^+^ and CD8^+^ T cells *ex vivo*^35^, and another program calculating TF affinity for DNA sequences highlighted RFX family members as most affected by rs138300818*.* Further functional work is needed to confirm RFX5/7 binding to the locus, and its direct effect on *THEMIS* (and *PTPRK*) in thymocytes, recent thymic emigrants, or other relevant cells.

Another limitation of this work is the sparsity of the thymocyte data. As only a few histone modification peaks were measured for each of the four individuals, and different histone modification combinations were available for each, it was not possible to define chromatin states within each individual. This was overcome by combining histone modification peaks across different biological replicates into pseudo-chromatin states, following a previously reported annotation, e.g., combining H3K4me1 and H3K27Ac signals for pseudostate 7, i.e., active enhancers^28^. While the lead findings at rs138300818 and in *AFF3* (rs66733041) were found for histone modification peaks, rs142852921 in *SKAP2* reached a significant difference in score only for the pseudostate 10, “distal active promoter”.

It should be noted that many of the identified thymocyte motifs were similar across the thymocyte cell types and histone modification peaks, which are not necessarily specific to thymocytes. This was reflected in the thymocyte motif comparison with known TF binding motifs, revealing 11 TFs, out of which most are expressed in nearly all tissues. Interestingly, SP3 has the highest expression in the thymus across the FANTOM5 tissues, supporting its relevance in thymocytes. Furthermore, the rs138300818 in the *PTPRK-THEMIS* locus was linked to thymocytes through chromatin conformation data from the thymus, and as the RFX7 was highly expressed in the thymus. While we hypothesized that the causal genetic variant in the 6q22.33 locus would affect a regulatory motif active in the thymocytes, our genome-wide search for such motifs may have missed some more specific regulatory motifs only found in the 6q22.33 region.

To conclude, the use of two parallel approaches and versatile biological data on thymocytes suggests that the rs138300818 insertion protects from early-onset T1D through affecting regulatory elements in thymocytes, including RFX5/7 binding. Functional studies are needed to confirm our findings.

## Methods

### Overall study design

We sought DNA motifs located in active chromosome regions of thymocyte cells, defined as histone modification peaks and overlapping RNA expression, indicative of active enhancer regions^25^. Resulting thymocyte motifs were integrated with the 58 credible set variants on the 6q22.33 locus, and 1,165 other SNPs for T1D, to identify SNPs that disrupt DNA motifs identified in the thymocyte epigenetic data. In addition, we applied a deep-learning method to identify credible set variants that alter TF binding motifs (Fig. 1).

### Study material

Genome-wide chromatin immunoprecipitation sequencing (ChIP-seq) data on histone modification marks were available from the Blueprint epigenomics project^26^ (accessed through ftp://ftp.ebi.ac.uk/pub/databases/blueprint/data/homo_sapiens/GRCh38/) for four individuals. Available peaks included H3K4 trimethylation (H3K4me3), H3K4 monomethylation (H3K4me1), H3K27 acetylation (H3K27ac), H3K36 trimethylation (H3K36me3), and H3K27 trimethylation (H3K27me3). Samples were dissected to CD3 ^− ^CD4^+^CD8^+^ thymocytes, CD3^+^CD4^+^CD8^+^ thymocytes, CD4^+^αβ thymocytes, and CD8^+^αβ thymocytes. Total RNA-seq data was available and downloaded for these cell types for one individual.

Human reference genome DNA sequence primary assembly and exon annotations were downloaded from Ensembl (ftp.ensembl.org/pub/release-92/) for human genome build GRCh38, release 92. DNA positions were updated from human genome build GRCh37 to GRCh38 using the UCSC liftover tool (https://genome.ucsc.edu/cgi-bin/hgLiftOver) when necessary.

SNPs associated with T1D were obtained from Burren et al.^31^, where credible set SNPs were defined as those with group posterior probability ≥ 0.9 from GUESSFM statistical fine mapping of ImmunoChip GWAS data, further filtered to a total of 1165 variants with *p*-value < 1 × 10^ − 5^ for association with T1D.

3D chromatin conformation capture data based on Promoter capture Hi-C (PCHiC) were obtained for 16 primary blood cell types and thymus^21^, CD34^+^ stem cells and human Epstein-Barr virus (EBV)-transformed lymphoblastoid cell line GM12878^44^, and for pancreatic islets^22^ using the CHiCP browser^45^. Interactions with CHiCAGO score > 5 were considered high-confidence^21^. eQTL associations were queried in the GTEX v8 (any tissue, gtexportal.org) and eQTLgen whole blood cis-eQTL databases^46^. We queried RNA expression data for the genes of interest from Human Protein Atlas (HPA) version 18, for HPA, Genotype-Tissue Expression (GTEx), and Functional Annotation of Mammalian Genomes 5 (FANTOM5) transcriptome data sets.

### Statistical analysis

#### Histone modification ChIP-seq peaks

A general overview of the thymocyte histone modification motif search and integration with the AAD and T1D SNPs is summarized in the Supplementary Fig. S4. First, bedtools v 2.27.1 software package^47^ and intersect tool were used for processing the histone modification peaks. Histone modification peaks were limited to those with peak fold change ≥ 3 and *q*-value < 0.0001. To enrich the search for active DNA sites, we limited the ChIP-seq peaks to those with total RNA-seq overlapping the peak, defined as mean RNA-seq depth ≥ 0.001 over the peak length, using UCSC bigWigSummary and bigWigAverageOverBed utilities (http://hgdownload.soe.ucsc.edu/admin/exe/). Finally, histone modification peaks of the same cell type, but from different biological repeats (individuals), were pooled together (Supplementary Table S2). Peak calls shorter than six base pairs (bp) were excluded. A flanking region of 20 bp was then added to allow TF binding motifs on the peak borders, and exonic regions were excluded to avoid capturing motifs for protein structural domains or other repetitive sequences found in exons.

We defined cell-type-specific chromatin pseudostates as an intersect of the obtained histone peaks using bedtools intersect tool, mimicking the previous definitions by Ernst and Kellis^48^ for pseudostate 7 (H3K4me1 and H3K36me3), pseudostate 9 (H3K4me1 and H3K27Ac), pseudostate 10 (H3K4me3, H3K27Ac, and H3K4me1), pseudostate 11 (H3K4me3 and H3K4me1), and pseudostate 12 (H3K4me3 and H3K27Ac), available for a subset of the thymocytes (Supplementary Table S3).

#### DNA Motif search

Fasta sequences were extracted for each peak with the bedtools getfasta tool. Motif discovery was performed using MEME Suite^27^ running MEME-ChIP, searching for 6–30 bp motifs enriched relative to a random first-order model (i.e. adjusting for nucleotide and dimer biases, e.g. CG content), targeting 20 motifs. As by default, 600 sequences were randomly selected for initial motif discovery. Significant motifs were defined as *E*-value < 0.01/17 cell type and histone mark combinations/20 motifs = 2.94 × 10^ − 5^. The resulting motifs were compared to the in vivo and in silico vertebrate DNA motifs (including JASPAR CORE vertebrates, UniProbe Mouse, and Jolma2013 Human and Mouse databases) with tomtom tool^27^; significant similarity was defined as q-value < 0.01.

#### Motif and sequence overlap

FASTA sequences were extracted for ± 30 bp around the 58 credible set SNPs for both the reference and alternative allele from Ensembl (build GRCh38) using R jsonlite package v.1.5^49^. Sequence overlap with the discovered thymocyte motifs was studied with the FIMO tool from the MEME suite^27^. Significant motif overlap was considered as P_REF_ or P_ALT_ < 0.01/253 significant thymocyte motifs (from MEME)/58 SNPs = 6.81 × 10^ − 7^. The thymocyte motif overlap was calculated similarly for SNPs in the credible sets associated with T1D^31^. Significant motif overlap was considered as P_REF_ or P_ALT_ < 0.01/253 significant thymocyte motifs/1,165 SNPs = 3.39 × 10^ − 8^.

We calculated an E-value E_diff_ (P_REF _− P_ALT_) for the difference in *p*-value between the REF and ALT alleles by calculating a z-score as (mean difference in *p*-values—difference in *p*-values)/(standard deviation of differences in *p*-values), and converting the z-score into an E-value (*p*-value) assuming two-tailed normal distribution. E_diff_ (Sc_REF _− Sc_ALT_) for the difference in score between the REF and ALT alleles was calculated in a similar manner. These scores are conservative, as only those SNP—motif pairs are considered where at least one of the SNP alleles had nominally significant (*p* < 0.05) similarity with a motif sequence. Distributions were calculated separately for each of the 17 cell-type—histone modification combinations, and E_diff_ (P_REF _− P_ALT_) and E_diff_ (Sc_REF _− Sc_ALT_) < 0.01/17 = 5.88 × 10^ − 4^ were considered significant.

#### Allele-specific TF binding sites

We employed DeepBind v0.11^29^ to assess allele-specific TF binding for the 58 SNPs in the credible set on the the 6q22.33 locus, as well as the two T1D SNPs affecting thymocyte motifs, comparing the 30 bp sequences flanking the SNPs. DeepBind was run using default parameters and all non-deprecated TFs delivered with the distribution (N = 927).

We additionally conducted transcription factor affinity prediction (TRAP) with sTRAP algorithm^30^ implemented in http://trap.molgen.mpg.de/cgi-bin/trap_two_seq_form.cgi for the same SNP sequeences. We used all JASPAR motifs contrasted to the human promoter background model, adjusted with the Benjamini–Hochberg method.

## Supplementary Information

Supplementary Information.

## Data Availability

All utilised data were downloaded from publicly available databases. The genome-wide chromatin immunoprecipitation sequencing (ChIP-seq) data on histone modification marks analysed during the current study are available in the Blueprint epigenomics project, through ftp://ftp.ebi.ac.uk/pub/databases/blueprint/data/homo_sapiens/GRCh38/. Human reference genome DNA sequence primary assembly and exon annotations for human genome build GRCh38, release 92, are available in the Ensembl repository (ftp.ensembl.org/pub/release-92/). All utilised software is freely available for research purposes.
