# Supplementary material for: Thymocyte regulatory variant alters transcription factor binding and protects from type 1 diabetes in infants
Source: Sci Rep. 2022 Aug 19;12:14137. doi: 10.1038/s41598-022-18296-4 (PMC9391468; doi:10.1038/s41598-022-18296-4)

**Supplementary Material**

**Thymocyte regulatory variant alters transcription factor binding and protects from type 1 diabetes in infants**

Sandholm Niina, Rubio García Arcadio, Pekalski Marcin L, Inshaw Jamie RJ, Cutler Antony J, Todd John A

[Supplementary Table S1. High-confidence chromatin 3D conformation capture interactions (CHiCAGO score > 5) in 16 primary blood cells and in foetal thymus for the 58 SNPs in the credible set of likely causal SNPs for age at diabetes diagnosis. 3](#_Toc109151588)

[Supplementary Table S2. Number and width of genome-wide histone modification peaks in raw data, after filtering, and in final analysis after pooling histone modification peaks from the same cell types, and after excluding exons. 5](#_Toc109151589)

[Supplementary Table S3. Number of peaks for combined chromatin pseudostates 6](#_Toc109151590)

[Supplementary Table S4. Overlap of discovered thymocyte motifs with known motifs 7](#_Toc109151591)

[Supplementary Table S5. Thymocyte motifs overlapping credible set SNPs with marked difference (two orders of magnitude in p-value) in motif-sequence similarity between the reference and alternative alleles.. 11](#_Toc109151592)

[Supplementary Table S6: DeepBind transcription factor binding motifs that are affected by T1D SNPs that also affect thymocyte motifs. 13](#_Toc109151593)

[Supplementary Table S7: Transcription factor binding affinity calculated with sTRAP for SNPs that also affect thymocyte motifs 14](#_Toc109151594)

[Supplementary Table S8. Thymocyte motifs overlapping T1D credible set SNPs with marked difference in motif-sequence similarity between the reference and alternative alleles. 15](#_Toc109151595)

[Supplementary Figure S1: THEMIS and PTPRK expression in single cell RNA sequencing of developing human immune system in thymus 19](#_Toc109151597)

[Supplementary Figure S2. Overlap between the discovered thymocyte motifs and known transcription factor (TF) binding motifs. 26](#_Toc109151598)

[Supplementary Figure S3. Predicted TF binding for all SNPs in credible set for age at diabetes diagnosis based on deepbind neural network predictions. 28](#_Toc109151599)

[Supplementary Figure S4: RFX7, RFX5, and RFX3 vs THEMIS expression in double negative (Q), double positive (Q), and αβ entry T cells, the cell types with the highest RFX7/5/3 expression in thymus in single cell RNA sequencing of developing human immune system (A,B,C). 29](#_Toc109151600)

[Supplementary Figure S5: Transcription factor (TF) binding affinity for the 58 SNPs on the chromosome 5p22.33 region, calculated with sTRAP software for all 1241 TFs with JASPAR matrices. 30](#_Toc109151601)

[Supplementary Figure S6. Non-coding RNA transcription overlapping rs142852921 (SKAP2). 31](#_Toc109151602)

[Supplementary Figure S7. Flow chart describing the analysis process for identifying thymocyte histone modification motifs and their integration with age-at-diabetes or T1D SNPs. 32](#_Toc109151603)

## Supplementary Table S1. High-confidence chromatin 3D conformation capture interactions (CHiCAGO score > 5) in 16 primary blood cells and in foetal thymus for the 58 SNPs in the credible set of likely causal SNPs for age at diabetes diagnosis. The variants are grouped under the PCHiC fragments that are of varying length, and the gene interaction results apply to all SNPs within the given fraction.

| **Fragment** | **Chr:pos_b37** | **SNP** | **Finemap** | **Tissue** | **Gene** | **ChiCAGO** |
| --- | --- | --- | --- | --- | --- | --- |
|  | 6:128265918 | rs802750 | 0.50 |  |  |  |
| *chr6:128,266,169..128,271,884 (5.71KB)* | 6:128266249 | rs6939352 | 0.96 | Foetal thymus | *PTPRK* | 8.18 |
|  | 6:128268564 | rs802747 | 0.50 | Naive CD4 | *PTPRK* | 5.28 |
|  | 6:128269179 | rs802746 | 0.42 |  |  |  |
|  | 6:128270066 | rs9491889 | 0.96 |  |  |  |
|  | 6:128270122 | rs9491890 | 0.96 |  |  |  |
| *chr6:128,271,885..128,279,291 (7.41KB)* | 6:128272323 | rs802744 | 0.50 | Foetal thymus | *PTPRK* | 7.6 |
|  | 6:128272875 | rs802743 | 0.50 |  |  |  |
|  | 6:128276699 | rs802740 | 0.50 |  |  |  |
|  | 6:128277150 | rs9491891 | 0.96 |  |  |  |
|  | 6:128277209 | rs802739 | 0.50 |  |  |  |
|  | 6:128277274 | rs147626184 | 0.96 |  |  |  |
|  | 6:128277932 | rs802738 | 0.50 |  |  |  |
|  | 6:128278052 | rs802737 | 0.50 |  |  |  |
|  | 6:128278121 | rs376827043 | 0.50 |  |  |  |
|  | 6:128278229 | rs1418600 | 0.50 |  |  |  |
|  | 6:128278230 | rs1418601 | 0.50 |  |  |  |
|  | 6:128278232 | rs118097399 | 0.96 |  |  |  |
|  | 6:128278335 | rs802735 | 0.50 |  |  |  |
|  | 6:128278797 | rs802734 | 0.42 |  |  |  |
|  | 6:128279184 | rs802733 | 0.50 |  |  |  |
| *chr6:128,279,292…128,279,651 (0.36KB)* | 6:128279421 | rs802732 | 0.50 |  |  |  |
|  | 6:128279428 | rs802731 | 0.42 |  |  |  |
|  | 6:128279496 | rs35576497 | 0.50 |  |  |  |
| *chr6:128,279,652..128,284,437 (4.79KB)* | 6:128280103 | rs802730 | 0.42 | Foetal thymus | *PTPRK* | 5.62 |
|  | 6:128280357 | rs9491892 | 0.96 |  |  |  |
|  | 6:128280374 | rs9482848 | 0.96 |  |  |  |
|  | 6:128280930 | rs9491893 | 0.96 |  |  |  |
|  | 6:128281555 | rs802728 | 0.50 |  |  |  |
|  | 6:128281660 | rs802727 | 0.50 |  |  |  |
|  | 6:128281860 | rs802726 | 0.50 |  |  |  |
|  | 6:128282028 | rs802725 | 0.42 |  |  |  |
|  | 6:128282757 | rs1089653 | 0.42 |  |  |  |
|  | 6:128282782 | rs1089652 | 0.50 |  |  |  |
|  | 6:128283192 | rs802724 | 0.50 |  |  |  |
|  | 6:128284218 | rs802722 | 0.50 |  |  |  |
| *chr6:128,284,438..128,288,435 (4.00KB)* | 6:128284770 | rs802721 | 0.50 | Foetal thymus | *PTPRK* | 5.12 |
|  | 6:128286300 | rs113297984 | 0.96 |  |  |  |
|  | 6:128286385 | rs72973797 | 0.96 |  |  |  |
|  | 6:128287157 | rs72973800 | 0.96 |  |  |  |
|  | 6:128287847 | rs761332 | 0.96 |  |  |  |
| *chr6:128,288,436..128,290,187 (1.75KB* | 6:128288535 | rs9482849 | 0.96 | Foetal thymus | *PTPRK* | 11.35 |
|  | 6:128289018 | rs802719 | 0.42 |  |  |  |
|  | 6:128289213 | rs12111314 | 0.96 |  |  |  |
| *chr6:128,290,677..128,298,505 (7.83KB)* | 6:128291198 | rs3190930 | 0.42 | Foetal thymus | *PTPRK* | 13.79 |
|  | 6:128291648 | rs41285280 | 0.42 | Naive CD8 | *THEMIS* | 5.65 |
|  | 6:128291680 | rs11753289 | 0.96 |  |  |  |
|  | 6:128292391 | rs4559105 | 0.42 |  |  |  |
|  | 6:128293505 | rs9482850 | 0.96 |  |  |  |
|  | 6:128293561 | rs55743914 | 0.42 |  |  |  |
|  | 6:128293633 | rs9482851 | 0.96 |  |  |  |
|  | 6:128293931 | rs72975913 | 0.96 |  |  |  |
|  | 6:128294054 | rs72975916 | 0.96 |  |  |  |
|  | 6:128294708 | rs35469349 | 0.42 |  |  |  |
|  | 6:128295501 | rs7738609 | 0.96 |  |  |  |
|  | 6:128297021 | rs138300818 | 0.96 |  |  |  |
|  | 6:128297603 | rs3901020 | 0.96 |  |  |  |
|  | 6:128297610 | rs4510698 | 0.96 |  |  |  |

Fragment: the PCHiC fragment used as the target in the experiment, including multiple SNPs. Chromosome position is given in b37 coordinates. Finemap: posterior probability of causal variant within the group. Gene: the gene transcription start site (TSS) used as bait in the experiment. ChiCAGO: ChiCAGO score for the PCHiC interaction in 16 primary blood cell types and thymus (Javierre BM, Burren OS, Wilder SP, Kreuzhuber R, Hill SM, Sewitz S, et al. Lineage-Specific Genome Architecture Links Enhancers and Non-coding Disease Variants to Target Gene Promoters. Cell. 2016;167: 1369-1384.e19.). Score ≥5 was considered significant. No interactions were detected in pancreatic islets (Miguel-Escalada I, Bonàs-Guarch S, Cebola I, Ponsa-Cobas J, Mendieta-Esteban J, Atla G, et al. Human pancreatic islet three-dimensional chromatin architecture provides insights into the genetics of type 2 diabetes. Nat Genet. 2019;51: 1137–1148. doi:10.1038/s41588-019-0457-0)

## Supplementary Table S2. Number and width of genome-wide histone modification peaks in raw data, after filtering, and in final analysis after pooling histone modification peaks from the same cell types, and after excluding exons.

|  |  |  | **Raw peak calls** | | | | |  | **Filtered peaks** | | | |  |  |
| --- | --- | --- | --- | --- | --- | --- | --- | --- | --- | --- | --- | --- | --- | --- |
| **Histone mod** | **Cell type** | **subj** | **N** | **Width** | **Min width** | **Max width** | **RNA %** |  | **N** | **Width** | **Min width** | **Max width** | **N peaks final** | **N motifs** |
| H3K4me3 | CD3^+^CD4^+^CD8^+^ | TH91 | 22407 | 910 | 150 | 30979 | 90 % |  | 18668 | 1104 | 150 | 30979 | 48295 | 17 |
|  |  | TH101 | 33636 | 887 | 180 | 36509 | 87 % |  | 25661 | 1252 | 180 | 36509 |  |  |
|  |  | TH118 | 34356 | 539 | 136 | 30024 | 87 % |  | 26972 | 792 | 136 | 30024 |  |  |
|  | CD3^-^CD4^+^CD8^+^ | TH89 | 18354 | 703 | 186 | 9758 | 92 % |  | 16125 | 787 | 186 | 9758 | 33132 | 19 |
|  |  | TH91 | 22947 | 722 | 130 | 13188 | 91 % |  | 19659 | 886 | 130 | 13188 |  |  |
|  | CD4^+^αß | TH91 | 26339 | 911 | 160 | 29319 | 84 % |  | 20448 | 1188 | 160 | 29319 | 37452 | 20 |
|  | CD8^+^αß | TH91 | 25161 | 1000 | 170 | 16113 | 85 % |  | 19319 | 1292 | 170 | 16113 | 36825 | 18 |
| H3K4me1 | CD3^+^CD4^+^CD8^+^ | TH101 | 55622 | 1400 | 220 | 108319 | 80 % |  | 17551 | 2783 | 220 | 108319 | 36308 | 2 |
| H3K27ac | CD3^+^CD4^+^CD8^+^ | TH91 | 14287 | 373 | 176 | 5688 | 100 % |  | 13029 | 395 | 176 | 5688 | 33131 | 18 |
|  |  | TH118 | 38531 | 250 | 140 | 6534 | 97 % |  | 30450 | 293 | 140 | 6534 |  |  |
|  | CD3^-^CD4^+^CD8^+^ | TH89 | 13035 | 394 | 200 | 5394 | 99 % |  | 11334 | 423 | 200 | 5394 | 12334 | 17 |
|  | CD4^+^αß | TH91 | 7088 | 313 | 176 | 3567 | 99 % |  | 6505 | 323 | 176 | 3567 | 6011 | 18 |
| H3K36me3 | CD3^+^CD4^+^CD8^+^ | TH101 | 36594 | 2143 | 220 | 185670 | 100 % |  | 5563 | 14425 | 232 | 185670 | 53760 | 1 |
|  |  | TH118 | 41108 | 1333 | 190 | 135740 | 100 % |  | 1416 | 6843,5 | 190 | 135740 |  |  |
| H3K27me3 | CD3^-^CD4^+^CD8^+^ | TH89 | 14696 | 1099 | 180 | 116374 | 58 % |  | 1240 | 1368 | 180 | 20301 | 2333 | 20 |

Raw peak calls: unmodified histone modification peak calls from Encode data; filtered peaks: peaks after filtering for those with fold change ≥3, q-value<0.0001, and some level of total RNA sequencing overlapping the peak, indicative of active chromatin region; N peaks final: number of peaks after pooling all peaks from the same cell type, and excluding exons (potentially leading to splitting a peak into multiple new peak segments). N motifs: Number of significant DNA motifs (E-value < 2.94×10^-5^) underlying each cell type specific histone modification peak, identified with MEME software.

## Supplementary Table S3. Number of peaks for combined chromatin pseudostates

| **Chromatin  state** | **combination of the  histone modification marks** | **Thymocyte cell type** | **Number  of peaks** | **Number  of motifs** |
| --- | --- | --- | --- | --- |
| state7 | H3K4me1 and H3K36me3 | CD3^+^CD4^+^CD8^+^ | 3524 | 9 |
| state9 | H3K4me1 and H3K27Ac | CD3^+^CD4^+^CD8^+^ | 18906 | 16 |
| state10 | H3K4me3, H3K27Ac, and H3K4me1 | CD3^+^CD4^+^CD8^+^ | 14433 | 14 |
| state11 | H3K4me3 and H3K4me1 | CD3^+^CD4^+^CD8^+^ | 28932 | 16 |
| state12 | H3K4me3 and H3K27Ac | CD3^+^CD4^+^CD8^+^ | 24341 | 15 |
| state12 | H3K4me3 and H3K27Ac | CD3^-^CD4^+^CD8^+^ | 10390 | 19 |
| state12 | H3K4me3 and H3K27Ac | CD4^+^αß | 5741 | 14 |

Number of motifs: Number of significant DNA motifs underlying each celltype specific composite histone state peak, identified with MEME software. E-value for statistical significance was adjusted for multiple testing, E-value<0.01/17 cell type – histone modification or state combinations/20 motifs searched for each combination=2.94×10^-5^

## Supplementary Table S4. Overlap of discovered thymocyte motifs with known motifs

| **Histone_cell_type** | **Thymocyte motif ID** | **Target ID** | **Target name** | **Data base** | **p-value** | **E-value** | **q-value** |
| --- | --- | --- | --- | --- | --- | --- | --- |
| **H3K4me3_CD8_ab** | **GGGGGCGGSGCGSGG** | **KLF16_DBD** | **KLF16** | **Jolma2013** | **8.17E-07** | **1.48E-03** | **9.77E-04** |
| H3K4me3_CD8_ab | GGGGGCGGSGCGSGG | MA0741.1 | KLF16 | Jaspar core | 8.17E-07 | 1.48E-03 | 9.77E-04 |
| H3K4me3_CD3n_CD4p_CD8p | CCCSSSCCCCGCCSCCGCCCSCSSCSCSCC | KLF16_DBD | KLF16 | Jolma2013 | 3.70E-06 | 6.69E-03 | 2.85E-03 |
| H3K4me3_CD3n_CD4p_CD8p | CCCSSSCCCCGCCSCCGCCCSCSSCSCSCC | MA0741.1 | KLF16 | Jaspar core | 3.70E-06 | 6.69E-03 | 2.85E-03 |
| H3K4me3_CD3n_CD4p_CD8p | GSGGCGGBGSGGGSVGGRGGCGGGGCSGGS | KLF16_DBD | KLF16 | Jolma2013 | 4.01E-06 | 7.25E-03 | 2.39E-03 |
| H3K4me3_CD3n_CD4p_CD8p | GSGGCGGBGSGGGSVGGRGGCGGGGCSGGS | MA0741.1 | KLF16 | Jaspar core | 4.01E-06 | 7.25E-03 | 2.39E-03 |
| H3K4me3_CD8_ab | GGGGGCGGGGCSGGGSGGSSSGGGSSSGSG | KLF16_DBD | KLF16 | Jolma2013 | 1.15E-05 | 2.08E-02 | 6.85E-03 |
| H3K4me3_CD8_ab | GGGGGCGGGGCSGGGSGGSSSGGGSSSGSG | MA0741.1 | KLF16 | Jaspar core | 1.15E-05 | 2.08E-02 | 6.85E-03 |
| **H3K4me3_CD8_ab** | **GGGGGCGGSGCGSGG** | **MA0599.1** | **KLF5** | **Jaspar core** | **3.39E-06** | **6.13E-03** | **2.43E-03** |
| H3K4me3_CD3p_CD4p_CD8p | CSCSCGCCCCSGCCCCGCCCC | MA0599.1 | KLF5 | Jaspar core | 6.54E-06 | 1.18E-02 | 7.79E-03 |
| H3K4me3_CD3n_CD4p_CD8p | GSGGCGGBGSGGGSVGGRGGCGGGGCSGGS | MA0599.1 | KLF5 | Jaspar core | 1.25E-05 | 2.26E-02 | 5.10E-03 |
| H3K4me3_CD3n_CD4p_CD8p | CCCSSSCCCCGCCSCCGCCCSCSSCSCSCC | MA0599.1 | KLF5 | Jaspar core | 1.72E-05 | 3.11E-02 | 6.85E-03 |
| H3K4me3_CD8_ab | GGGGGCGGGGCSGGGSGGSSSGGGSSSGSG | MA0599.1 | KLF5 | Jaspar core | 2.16E-05 | 3.90E-02 | 9.11E-03 |
| **H3K4me3_CD3n_CD4p_CD8p** | **GSGGCGGBGSGGGSVGGRGGCGGGGCSGGS** | **MA0079.3** | **SP1** | **Jaspar core** | **4.01E-08** | **7.26E-05** | **7.19E-05** |
| H3K4me3_CD8_ab | GGGGGCGGSGCGSGG | MA0079.3 | SP1 | Jaspar core | 8.08E-08 | 1.46E-04 | 2.90E-04 |
| H3K4me3_CD8_ab | GGGGGCGGGGCSGGGSGGSSSGGGSSSGSG | MA0079.3 | SP1 | Jaspar core | 9.05E-08 | 1.64E-04 | 2.23E-04 |
| H3K4me3_CD3n_CD4p_CD8p | CCCSSSCCCCGCCSCCGCCCSCSSCSCSCC | MA0079.3 | SP1 | Jaspar core | 2.84E-07 | 5.14E-04 | 5.09E-04 |
| H3K27ac_CD4_ab | CSSSCSSCGCCCCSGCCSCSS | MA0079.3 | SP1 | Jaspar core | 4.15E-07 | 7.50E-04 | 7.42E-04 |
| H3K27ac_CD3n_CD4p_CD8p | CSCSSCCSSCCSCBCCCCCCSSCCCBSSSC | MA0079.3 | SP1 | Jaspar core | 1.23E-06 | 2.22E-03 | 2.20E-03 |
| H3K4me3_CD3n_CD4p_CD8p | GSGGSGGGSGGSGSGGGCGSGGSGSGSGGS | MA0079.3 | SP1 | Jaspar core | 1.43E-06 | 2.58E-03 | 1.73E-03 |
| H3K4me3_CD3p_CD4p_CD8p | CCSSSCCCHSRSCCCSCCCCSSCCSCSCSS | MA0079.3 | SP1 | Jaspar core | 1.49E-06 | 2.69E-03 | 2.26E-03 |
| H3K4me3_CD4_ab | CSSCGCCCCCKCCCCSGCSCBSCSCCBCCS | MA0079.3 | SP1 | Jaspar core | 1.61E-06 | 2.90E-03 | 2.88E-03 |
| H3K4me3_CD4_ab | CSCSCCCCSCSCCGCCCCCGCCCSCSCSC | MA0079.3 | SP1 | Jaspar core | 1.73E-06 | 3.12E-03 | 3.09E-03 |
| H3K27ac_CD4_ab | CCCCCGCCCCSCCCCSSCCSSCSCCGCCS | MA0079.3 | SP1 | Jaspar core | 1.75E-06 | 3.16E-03 | 2.09E-03 |
| H3K4me3_CD4_ab | CSSSSCSCSCSCCSSCCCCGCCSCSSCCS | MA0079.3 | SP1 | Jaspar core | 2.58E-06 | 4.66E-03 | 4.61E-03 |
| H3K4me3_CD8_ab | GGGGSSGGGGCGGGGGCGSGG | MA0079.3 | SP1 | Jaspar core | 3.95E-06 | 7.15E-03 | 7.08E-03 |
| H3K4me3_CD8_ab | GGGGGCGGSGCGSGG | SP1_DBD | SP1 | Jolma2013 | 4.17E-06 | 7.55E-03 | 2.49E-03 |
| H3K27ac_CD3p_CD4p_CD8p | GGGGGDSGRGGMGGRGGSRGG | MA0079.3 | SP1 | Jaspar core | 4.22E-06 | 7.64E-03 | 3.80E-03 |
| H3K4me3_CD3p_CD4p_CD8p | GSSSGGGGCGGSGGCGSSGGS | MA0079.3 | SP1 | Jaspar core | 4.28E-06 | 7.73E-03 | 7.65E-03 |
| H3K4me3_CD3p_CD4p_CD8p | CSCSCGCCCCSGCCCCGCCCC | MA0079.3 | SP1 | Jaspar core | 4.38E-06 | 7.91E-03 | 7.79E-03 |
| H3K4me3_CD3p_CD4p_CD8p | SGGSSSGGGSCGGGVGCGGVGSSGSSSGSG | MA0079.3 | SP1 | Jaspar core | 4.60E-06 | 8.31E-03 | 5.49E-03 |
| H3K27ac_CD3p_CD4p_CD8p | GSSSSSGSGGSGGMGSGGGSSGGGSCGGGG | MA0079.3 | SP1 | Jaspar core | 4.79E-06 | 8.65E-03 | 5.73E-03 |
| H3K4me3_CD3n_CD4p_CD8p | GSGGCGGBGSGGGSVGGRGGCGGGGCSGGS | SP1_DBD | SP1 | Jolma2013 | 1.16E-05 | 2.11E-02 | 5.10E-03 |
| H3K4me3_CD8_ab | SGSSSGSSGSGGSSGSGGCNGGGSGSGGGG | MA0079.3 | SP1 | Jaspar core | 1.31E-05 | 2.37E-02 | 9.37E-03 |
| H3K4me3_CD8_ab | GGGGGCGGGGCSGGGSGGSSSGGGSSSGSG | SP1_DBD | SP1 | Jolma2013 | 2.46E-05 | 4.46E-02 | 9.11E-03 |
| **H3K4me3_CD3n_CD4p_CD8p** | **GSGGCGGBGSGGGSVGGRGGCGGGGCSGGS** | **MA0516.1** | **SP2** | **Jaspar core** | **3.69E-10** | **6.68E-07** | **1.32E-06** |
| H3K4me3_CD3p_CD4p_CD8p | CCSSSCCCHSRSCCCSCCCCSSCCSCSCSS | MA0516.1 | SP2 | Jaspar core | 4.36E-09 | 7.88E-06 | 1.56E-05 |
| H3K4me3_CD3n_CD4p_CD8p | CCCSSSCCCCGCCSCCGCCCSCSSCSCSCC | MA0516.1 | SP2 | Jaspar core | 8.69E-09 | 1.57E-05 | 3.11E-05 |
| H3K27ac_CD3n_CD4p_CD8p | CSCSSCCSSCCSCBCCCCCCSSCCCBSSSC | MA0516.1 | SP2 | Jaspar core | 1.54E-08 | 2.79E-05 | 5.52E-05 |
| H3K27ac_CD4_ab | CCCCCGCCCCSCCCCSSCCSSCSCCGCCS | MA0516.1 | SP2 | Jaspar core | 2.08E-08 | 3.76E-05 | 7.46E-05 |
| H3K4me3_CD4_ab | CSSSSCSCSCSCCSSCCCCGCCSCSSCCS | MA0516.1 | SP2 | Jaspar core | 2.89E-08 | 5.23E-05 | 1.03E-04 |
| H3K27ac_CD4_ab | CSSSCSSCGCCCCSGCCSCSS | MA0516.1 | SP2 | Jaspar core | 3.65E-08 | 6.59E-05 | 1.31E-04 |
| H3K27ac_CD3p_CD4p_CD8p | GGGGGDSGRGGMGGRGGSRGG | MA0516.1 | SP2 | Jaspar core | 4.20E-08 | 7.60E-05 | 7.55E-05 |
| H3K4me3_CD3p_CD4p_CD8p | SGGSSSGGGSCGGGVGCGGVGSSGSSSGSG | MA0516.1 | SP2 | Jaspar core | 4.32E-08 | 7.82E-05 | 1.55E-04 |
| H3K4me3_CD8_ab | SGSSSGSSGSGGSSGSGGCNGGGSGSGGGG | MA0516.1 | SP2 | Jaspar core | 5.12E-08 | 9.26E-05 | 1.83E-04 |
| H3K4me3_CD3n_CD4p_CD8p | GSGGSGGGSGGSGSGGGCGSGGSGSGSGGS | MA0516.1 | SP2 | Jaspar core | 5.12E-08 | 9.25E-05 | 1.84E-04 |
| H3K27ac_CD3p_CD4p_CD8p | GSSSSSGSGGSGGMGSGGGSSGGGSCGGGG | MA0516.1 | SP2 | Jaspar core | 6.01E-08 | 1.09E-04 | 2.15E-04 |
| H3K4me3_CD8_ab | GGGGSSGGGGCGGGGGCGSGG | MA0516.1 | SP2 | Jaspar core | 6.04E-08 | 1.09E-04 | 2.17E-04 |
| H3K4me3_CD4_ab | CSCSCCCCSCSCCGCCCCCGCCCSCSCSC | MA0516.1 | SP2 | Jaspar core | 9.84E-08 | 1.78E-04 | 3.53E-04 |
| H3K4me3_CD8_ab | GGGGGCGGGGCSGGGSGGSSSGGGSSSGSG | MA0516.1 | SP2 | Jaspar core | 1.24E-07 | 2.25E-04 | 2.23E-04 |
| H3K4me3_CD4_ab | CSSCGCCCCCKCCCCSGCSCBSCSCCBCCS | MA0516.1 | SP2 | Jaspar core | 2.22E-07 | 4.02E-04 | 7.96E-04 |
| H3K27ac_CD3n_CD4p_CD8p | GGSGSSSGGGGSSSSGGMSGSGGSCGGSSG | MA0516.1 | SP2 | Jaspar core | 3.58E-07 | 6.48E-04 | 1.29E-03 |
| H3K4me3_CD3p_CD4p_CD8p | CSCSCGCCCCSGCCCCGCCCC | MA0516.1 | SP2 | Jaspar core | 5.13E-07 | 9.28E-04 | 1.84E-03 |
| H3K27ac_CD3p_CD4p_CD8p | SCSSSSCCSSCSCCSCCSCCGCSGCCSCS | MA0516.1 | SP2 | Jaspar core | 7.30E-07 | 1.32E-03 | 2.61E-03 |
| H3K27ac_CD3n_CD4p_CD8p | GGGSSGGGSSGGGSGSGGCGGSGGCSGCGG | MA0516.1 | SP2 | Jaspar core | 8.10E-07 | 1.46E-03 | 2.90E-03 |
| H3K4me3_CD4_ab | CSSCGCSCSCSGCCCCSSCSC | MA0516.1 | SP2 | Jaspar core | 8.27E-07 | 1.49E-03 | 2.94E-03 |
| H3K4me3_CD3p_CD4p_CD8p | GSSSGGGGCGGSGGCGSSGGS | MA0516.1 | SP2 | Jaspar core | 1.20E-06 | 2.17E-03 | 4.29E-03 |
| H3K27me3_CD3n_CD4p_CD8p | CGCSGCCSCCGCCSCCSCCSC | MA0516.1 | SP2 | Jaspar core | 1.33E-06 | 2.40E-03 | 4.76E-03 |
| H3K4me3_CD8_ab | GGGGGCGGSGCGSGG | MA0516.1 | SP2 | Jaspar core | 1.42E-06 | 2.57E-03 | 1.27E-03 |
| H3K27ac_CD3p_CD4p_CD8p | SGCGSGGCGGGCSSGGSCSGGGGGCSSGGG | MA0516.1 | SP2 | Jaspar core | 2.59E-06 | 4.69E-03 | 9.24E-03 |
| **H3K4me3_CD8_ab** | **GGGGGCGGSGCGSGG** | **MA0746.1** | **SP3** | **Jaspar core** | **6.78E-06** | **1.23E-02** | **3.04E-03** |
| H3K4me3_CD8_ab | GGGGGCGGSGCGSGG | SP3_DBD | SP3 | Jolma2013 | 6.78E-06 | 1.23E-02 | 3.04E-03 |
| H3K4me3_CD3n_CD4p_CD8p | CCCSSSCCCCGCCSCCGCCCSCSSCSCSCC | MA0746.1 | SP3 | Jaspar core | 1.22E-05 | 2.21E-02 | 5.47E-03 |
| H3K4me3_CD3n_CD4p_CD8p | CCCSSSCCCCGCCSCCGCCCSCSSCSCSCC | SP3_DBD | SP3 | Jolma2013 | 1.22E-05 | 2.21E-02 | 5.47E-03 |
| H3K4me3_CD3n_CD4p_CD8p | GSGGCGGBGSGGGSVGGRGGCGGGGCSGGS | MA0746.1 | SP3 | Jaspar core | 1.57E-05 | 2.84E-02 | 5.12E-03 |
| H3K4me3_CD3n_CD4p_CD8p | GSGGCGGBGSGGGSVGGRGGCGGGGCSGGS | SP3_DBD | SP3 | Jolma2013 | 1.57E-05 | 2.84E-02 | 5.12E-03 |
| **H3K4me3_CD3n_CD4p_CD8p** | **GSGGCGGBGSGGGSVGGRGGCGGGGCSGGS** | **UP00002_1** | **Sp4** | **uniprobe_mouse** | **4.49E-07** | **8.12E-04** | **5.36E-04** |
| H3K4me3_CD3n_CD4p_CD8p | CCCSSSCCCCGCCSCCGCCCSCSSCSCSCC | UP00002_1 | Sp4 | uniprobe_mouse | 4.78E-06 | 8.65E-03 | 2.85E-03 |
| **H3K27ac_CD4_ab** | **CCCCCGCCCCSCCCCSSCCSSCSCCGCCS** | **UP00021_1** | **Zfp281 (Znf281 )** | **uniprobe_mouse** | **2.75E-07** | **4.97E-04** | **4.93E-04** |
| H3K27ac_CD3p_CD4p_CD8p | GGGGGDSGRGGMGGRGGSRGG | UP00021_1 | Zfp281 (Znf281 ) | uniprobe_mouse | 3.13E-07 | 5.65E-04 | 3.75E-04 |
| H3K4me3_CD8_ab | GGGGGCGGGGCSGGGSGGSSSGGGSSSGSG | UP00021_1 | Zfp281 (Znf281 ) | uniprobe_mouse | 9.61E-07 | 1.74E-03 | 1.15E-03 |
| H3K4me3_CD3n_CD4p_CD8p | GSGGSGGGSGGSGSGGGCGSGGSGSGSGGS | UP00021_1 | Zfp281 (Znf281 ) | uniprobe_mouse | 1.44E-06 | 2.61E-03 | 1.73E-03 |
| H3K27ac_CD4_ab | RGGRRRGGGRAGGRRARRRGR | UP00021_1 | Zfp281 (Znf281 ) | uniprobe_mouse | 1.51E-06 | 2.72E-03 | 2.72E-03 |
| H3K4me3_CD3p_CD4p_CD8p | CCSSSCCCHSRSCCCSCCCCSSCCSCSCSS | UP00021_1 | Zfp281 (Znf281 ) | uniprobe_mouse | 1.89E-06 | 3.41E-03 | 2.26E-03 |
| H3K4me3_CD3p_CD4p_CD8p | SGGSSSGGGSCGGGVGCGGVGSSGSSSGSG | UP00021_1 | Zfp281 (Znf281 ) | uniprobe_mouse | 1.96E-06 | 3.55E-03 | 3.51E-03 |
| H3K4me3_CD3n_CD4p_CD8p | GSGGCGGBGSGGGSVGGRGGCGGGGCSGGS | UP00021_1 | Zfp281 (Znf281 ) | uniprobe_mouse | 2.56E-06 | 4.62E-03 | 2.29E-03 |
| H3K4me3_CD3n_CD4p_CD8p | YCTCCCTCYCYCYCYYYCTCY | UP00021_1 | Zfp281 (Znf281 ) | uniprobe_mouse | 3.11E-06 | 5.62E-03 | 5.62E-03 |
| H3K27ac_CD3p_CD4p_CD8p | SCSSSSCCSSCSCCSCCSCCGCSGCCSCS | UP00021_1 | Zfp281 (Znf281 ) | uniprobe_mouse | 3.54E-06 | 6.40E-03 | 6.34E-03 |
| H3K27ac_CD3n_CD4p_CD8p | GGSGSSSGGGGSSSSGGMSGSGGSCGGSSG | UP00021_1 | Zfp281 (Znf281 ) | uniprobe_mouse | 3.92E-06 | 7.09E-03 | 7.04E-03 |
| H3K4me3_CD3n_CD4p_CD8p | CCCSSSCCCCGCCSCCGCCCSCSSCSCSCC | UP00021_1 | Zfp281 (Znf281 ) | uniprobe_mouse | 4.08E-06 | 7.38E-03 | 2.85E-03 |
| H3K4me3_CD4_ab | CSSCGCCCCCKCCCCSGCSCBSCSCCBCCS | UP00021_1 | Zfp281 (Znf281 ) | uniprobe_mouse | 4.75E-06 | 8.59E-03 | 5.68E-03 |
| H3K4me3_CD8_ab | GGGGSSGGGGCGGGGGCGSGG | UP00021_1 | Zfp281 (Znf281 ) | uniprobe_mouse | 6.85E-06 | 1.24E-02 | 8.19E-03 |
| H3K4me3_CD4_ab | CSCSCCCCSCSCCGCCCCCGCCCSCSCSC | UP00021_1 | Zfp281 (Znf281 ) | uniprobe_mouse | 7.04E-06 | 1.27E-02 | 8.41E-03 |
| H3K27ac_CD3n_CD4p_CD8p | CSCSSCCSSCCSCBCCCCCCSSCCCBSSSC | UP00021_1 | Zfp281 (Znf281 ) | uniprobe_mouse | 7.80E-06 | 1.41E-02 | 9.31E-03 |
| H3K27ac_CD4_ab | CSSSCSSCGCCCCSGCCSCSS | UP00021_1 | Zfp281 (Znf281 ) | uniprobe_mouse | 8.13E-06 | 1.47E-02 | 9.71E-03 |
| H3K4me3_CD8_ab | SGSSSGSSGSGGSSGSGGCNGGGSGSGGGG | UP00021_1 | Zfp281 (Znf281 ) | uniprobe_mouse | 8.99E-06 | 1.63E-02 | 8.02E-03 |
| H3K27ac_CD3p_CD4p_CD8p | GSSSSSGSGGSGGMGSGGGSSGGGSCGGGG | UP00021_1 | Zfp281 (Znf281 ) | uniprobe_mouse | 9.27E-06 | 1.68E-02 | 6.63E-03 |
| H3K4me3_CD8_ab | GGGGGCGGSGCGSGG | UP00021_1 | Zfp281 (Znf281 ) | uniprobe_mouse | 2.03E-05 | 3.67E-02 | 8.09E-03 |
| **H3K4me3_CD8_ab** | **GGGGGCGGGGCSGGGSGGSSSGGGSSSGSG** | **UP00022_1** | **Zfp740 (Znf740)** | **uniprobe_mouse** | **2.42E-05** | **4.37E-02** | **9.11E-03** |
| **H3K4me3_CD8_ab** | **SGSSSGSSGSGGSSGSGGCNGGGSGSGGGG** | **MA0146.2** | **Zfx** | **Jaspar core Mouse** | **2.34E-06** | **4.23E-03** | **4.17E-03** |
| H3K27ac_CD3p_CD4p_CD8p | GSSSSSGSGGSGGMGSGGGSSGGGSCGGGG | MA0146.2 | Zfx | Jaspar core Mouse | 4.81E-06 | 8.70E-03 | 5.73E-03 |
| H3K4me3_CD3n_CD4p_CD8p | GSGGCGGBGSGGGSVGGRGGCGGGGCSGGS | MA0146.2 | Zfx | Jaspar core Mouse | 1.28E-05 | 2.32E-02 | 5.10E-03 |
| H3K4me3_CD8_ab | GGGGGCGGGGCSGGGSGGSSSGGGSSSGSG | MA0146.2 | Zfx | Jaspar core Mouse | 2.54E-05 | 4.60E-02 | 9.11E-03 |
| **H3K4me3_CD3n_CD4p_CD8p** | **YCTCCCTCYCYCYCYYYCTCY** | **MA0528.1** | **ZNF263** | **Jaspar core** | **3.74E-11** | **6.76E-08** | **1.35E-07** |
| H3K27ac_CD3p_CD4p_CD8p | GGGGGDSGRGGMGGRGGSRGG | MA0528.1 | ZNF263 | Jaspar core | 1.67E-09 | 3.02E-06 | 6.00E-06 |
| H3K27ac_CD4_ab | RGGRRRGGGRAGGRRARRRGR | MA0528.1 | ZNF263 | Jaspar core | 2.16E-09 | 3.90E-06 | 7.79E-06 |
| H3K4me3_CD8_ab | YCTCYCTCYCYCTCY | MA0528.1 | ZNF263 | Jaspar core | 1.73E-07 | 3.12E-04 | 6.25E-04 |
| H3K27ac_CD4_ab | CCTCCTCYYCC | MA0528.1 | ZNF263 | Jaspar core | 3.39E-07 | 6.13E-04 | 1.23E-03 |
| H3K27ac_CD3p_CD4p_CD8p | GAGRGRARGGRRRARRGARAA | MA0528.1 | ZNF263 | Jaspar core | 1.85E-06 | 3.34E-03 | 6.68E-03 |
| H3K4me3_CD4_ab | TCCTCCTCCYC | MA0528.1 | ZNF263 | Jaspar core | 1.98E-06 | 3.58E-03 | 7.16E-03 |
| H3K4me3_CD8_ab | GGGGGCGGGGCSGGGSGGSSSGGGSSSGSG | MA0528.1 | ZNF263 | Jaspar core | 1.02E-05 | 1.84E-02 | 6.85E-03 |
| H3K4me3_CD3n_CD4p_CD8p | GSGGCGGBGSGGGSVGGRGGCGGGGCSGGS | MA0528.1 | ZNF263 | Jaspar core | 1.72E-05 | 3.11E-02 | 5.14E-03 |
|  |  |  |  |  |  |  |  |
| **state12_CD3n_CD4p_CD8p** | **CCCCCRYCCCCNCCCMCACCC** | **MA0073.1** | **RREB1** | **Jaspar core** | **1.07E-06** | **1.93E-03** | **3.85E-03** |
| state12_CD3p_CD4p_CD8p | GGGGSGSSCGGGGCGGSGSVGGSSSCGGG | MA0079.3 | SP1 | Jaspar core | 1.51E-06 | 2.72E-03 | 2.70E-03 |
| state12_CD3n_CD4p_CD8p | SCSSSCSSCGCCSCCSSCSCC | MA0079.3 | SP1 | Jaspar core | 1.71E-06 | 3.09E-03 | 3.04E-03 |
| state12_CD3p_CD4p_CD8p | SCSSCSSCGCCSSCCCCGCCCCSSCCCSC | MA0079.3 | SP1 | Jaspar core | 3.31E-06 | 5.99E-03 | 5.93E-03 |
| state12_CD3n_CD4p_CD8p | GSSGGSGSGGGGSSGGGCGGGGSGCGSGSS | MA0079.3 | SP1 | Jaspar core | 3.64E-06 | 6.59E-03 | 4.35E-03 |
| state11_CD3p_CD4p_CD8p | GSGSSSGGCSGGGGGSGGRGSSGGSGGSSG | MA0079.3 | SP1 | Jaspar core | 3.90E-06 | 7.05E-03 | 4.66E-03 |
| state9_CD3p_CD4p_CD8p | SSGGSSSGGSCGGGGSCGGGGGBSGSSGG | MA0079.3 | SP1 | Jaspar core | 5.18E-06 | 9.36E-03 | 8.16E-03 |
| state12_CD4_ab | CSCCGCSSSCSCCSSCSCCSCCSSSSCCGC | MA0079.3 | SP1 | Jaspar core | 6.37E-06 | 1.15E-02 | 8.85E-03 |
| state11_CD3p_CD4p_CD8p | GSGSSSGGCSGGGGGSGGRGSSGGSGGSSG | MA0516.1 | SP2 | Jaspar core | 3.42E-08 | 6.19E-05 | 1.23E-04 |
| state12_CD3n_CD4p_CD8p | GSSGGSGSGGGGSSGGGCGGGGSGCGSGSS | MA0516.1 | SP2 | Jaspar core | 4.22E-08 | 7.64E-05 | 1.51E-04 |
| state12_CD4_ab | CSCCGCSSSCSCCSSCSCCSCCSSSSCCGC | MA0516.1 | SP2 | Jaspar core | 1.36E-07 | 2.46E-04 | 4.86E-04 |
| state12_CD3p_CD4p_CD8p | SCSSCSSCGCCSSCCCCGCCCCSSCCCSC | MA0516.1 | SP2 | Jaspar core | 1.58E-07 | 2.86E-04 | 5.66E-04 |
| state12_CD3p_CD4p_CD8p | GGGGSGSSCGGGGCGGSGSVGGSSSCGGG | MA0516.1 | SP2 | Jaspar core | 2.33E-07 | 4.21E-04 | 8.34E-04 |
| state11_CD3p_CD4p_CD8p | CSCSSSCCCSSSCSCSCSCCSSCCSCGCSC | MA0516.1 | SP2 | Jaspar core | 2.40E-07 | 4.33E-04 | 8.59E-04 |
| state12_CD3n_CD4p_CD8p | SCSSSCSSCGCCSCCSSCSCC | MA0516.1 | SP2 | Jaspar core | 2.75E-07 | 4.96E-04 | 9.75E-04 |
| state12_CD4_ab | CCSSCSSCSCCCCSGCCSCCBCCCKSSCC | MA0516.1 | SP2 | Jaspar core | 2.84E-07 | 5.14E-04 | 1.02E-03 |
| state9_CD3p_CD4p_CD8p | SSGGSSSGGSCGGGGSCGGGGGBSGSSGG | MA0516.1 | SP2 | Jaspar core | 2.94E-07 | 5.32E-04 | 1.05E-03 |
| state10_CD3p_CD4p_CD8p | CCGCSCSCSCCSSSSCSCCCSGCCCCSSSC | MA0516.1 | SP2 | Jaspar core | 1.58E-06 | 2.85E-03 | 5.65E-03 |
| state12_CD3n_CD4p_CD8p | CCCCCRYCCCCNCCCMCACCC | MA0516.1 | SP2 | Jaspar core | 5.30E-06 | 9.59E-03 | 6.36E-03 |
| state12_CD3p_CD4p_CD8p | GGGGAWGGRGGAGGG | UP00002_1 | Sp4 | uniprobe_mouse | 1.91E-06 | 3.46E-03 | 3.45E-03 |
| state12_CD3n_CD4p_CD8p | GSSGGSGSGGGGSSGGGCGGGGSGCGSGSS | UP00021_1 | Zfp281 (Znf281 ) | uniprobe_mouse | 1.12E-06 | 2.02E-03 | 2.00E-03 |
| state11_CD3p_CD4p_CD8p | CSCSSSCCCSSSCSCSCSCCSSCCSCGCSC | UP00021_1 | Zfp281 (Znf281 ) | uniprobe_mouse | 1.35E-06 | 2.44E-03 | 2.42E-03 |
| state11_CD3p_CD4p_CD8p | GSGSSSGGCSGGGGGSGGRGSSGGSGGSSG | UP00021_1 | Zfp281 (Znf281 ) | uniprobe_mouse | 2.73E-06 | 4.93E-03 | 4.66E-03 |
| state12_CD4_ab | CCSSCSSCSCCCCSGCCSCCBCCCKSSCC | UP00021_1 | Zfp281 (Znf281 ) | uniprobe_mouse | 2.82E-06 | 5.09E-03 | 5.05E-03 |
| state12_CD3n_CD4p_CD8p | CCCCCRYCCCCNCCCMCACCC | UP00021_1 | Zfp281 (Znf281 ) | uniprobe_mouse | 5.30E-06 | 9.59E-03 | 6.36E-03 |
| state12_CD4_ab | CSCCGCSSSCSCCSSCSCCSCCSSSSCCGC | UP00021_1 | Zfp281 (Znf281 ) | uniprobe_mouse | 7.42E-06 | 1.34E-02 | 8.85E-03 |
| state9_CD3p_CD4p_CD8p | SSGGSSSGGSCGGGGSCGGGGGBSGSSGG | MA0146.2 | Zfx | Jaspar core Mouse | 6.84E-06 | 1.24E-02 | 8.16E-03 |
| state12_CD3p_CD4p_CD8p | CTTYCYCYCTCYCYYYCYCYTCCTCYYHCY | MA0528.1 | ZNF263 | Jaspar core | 1.06E-10 | 1.91E-07 | 3.82E-07 |
| state12_CD3n_CD4p_CD8p | GGRGGAGGRGGAGGR | MA0528.1 | ZNF263 | Jaspar core | 5.02E-10 | 9.07E-07 | 1.81E-06 |
| state9_CD3p_CD4p_CD8p | CCCTCYYYCTCYYCC | MA0528.1 | ZNF263 | Jaspar core | 3.73E-09 | 6.74E-06 | 1.35E-05 |
| state12_CD4_ab | KGRGGRWGGGGRAGSRGRGRV | MA0528.1 | ZNF263 | Jaspar core | 1.09E-08 | 1.97E-05 | 3.94E-05 |
| state12_CD3p_CD4p_CD8p | GGGGAWGGRGGAGGG | MA0528.1 | ZNF263 | Jaspar core | 4.68E-07 | 8.47E-04 | 1.69E-03 |
| state12_CD4_ab | CTYTYYCTCYTCCTY | MA0528.1 | ZNF263 | Jaspar core | 6.78E-07 | 1.23E-03 | 2.45E-03 |

A total of 48 motifs (135 thymocyte motif – transcription factor (TF) binding motif combinations) were significantly similar with q<0.01. Target ID/name: Known TF ID/name. Data base: data base for the known TF motifs, human unless otherwise stated. P/E/Q-values: statistical significance for motif similarity between the thymocyte motif and known TF binding motifs. For each known TF, the thymocyte motif with lowest q-value is highlighted in bold.

## Supplementary **Table S5. Thymocyte motifs overlapping credible set SNPs with marked difference (two orders of magnitude in p-value) in motif-sequence similarity between the reference and alternative alleles.** Significant motif overlap was considered as *P*_REF_ or *P*_ALT_ < 0.01/253 thymocyte motifs/58 SNPs = 6.81×10^-7^. Significant allelic difference was calculated based on observed distribution of p-value and score differences, separately for each of the 17 histone mark – cell type combinations and defined as E_diff_(P_REF_-P_ALT_) and E_diff_(Sc_REF_‑Sc_ALT_) < 0.01/17 = 5.88×10­^‑4^, respectively. Significant values are indicated with bold.

| **SNP** | **matched_sequence.REF/ALT** | **REF** | **ALT** | **hmark** | **Cell type** | **P_REF_** | **P_ALT_** | **Sc_REF_** | **Sc_ALT_** | **E_Diff_**  **(P_REF_ - P_ALT_)** | **E_Diff_**  **(Sc_REF_ - Sc_ALT_)** |
| --- | --- | --- | --- | --- | --- | --- | --- | --- | --- | --- | --- |
| rs138300818 | ATGGGGTTTCACCACGTTG-/GCCAGGCTGGTC |  | G | H3K27ac | CD4_ab | 0.000112 | ***2.53E-13*** | -17.9 | 38.7 | ***2.51E-29*** | ***6.57E-07*** |
| rs138300818 | GGGGTTTCACCACGTTG-/GCCGGCTGGTCT |  | G | state12 | CD4_ab | 4.73E-05 | ***2.26E-12*** | -10.4 | 35.5 | ***2.89E-25*** | ***4.68E-05*** |
| rs138300818 | AAGACCAGCCTGG-/CCAACGTGGTGAAACCCC |  | G | H3K27ac | CD3n_CD4p_CD8p | 1.41E-05 | ***1.32E-13*** | -9.0 | 39.5 | ***1.56E-24*** | 1.79E-02 |
| rs138300818 | GGAGTTCAAGACCAGCCTGG-/CCAACG |  | G | state9 | CD3p_CD4p_CD8p | ***1.65E-08*** | ***5.58E-15*** | 17.0 | 43.4 | ***6.32E-23*** | 6.75E-02 |
| rs138300818 | GTTTCACCACGTTG-/GCCGGCTGGTCTTG |  | G | H3K4me3 | CD3n_CD4p_CD8p | 0.000333 | ***4.94E-11*** | -15.6 | 30.7 | ***1.59E-20*** | 1.65E-02 |
| rs138300818 | ACCACGTTG-/GCCGGCTGGTCT |  | G | H3K4me3 | CD3p_CD4p_CD8p | 4.86E-05 | ***6.49E-12*** | -9.0 | 34.4 | ***2.77E-20*** | 5.49E-03 |
| rs138300818 | CAGGAGTTCAAGACCAGCCTGG-/CCAACGTG |  | G | H3K27me3 | CD3n_CD4p_CD8p | ***2E-09*** | ***7.88E-16*** | 20.8 | 45.9 | ***5.51E-18*** | 6.70E-02 |
| rs138300818 | ACCAGCCTGG-/CCAACGTGGTGAAACCCCATC |  | G | H3K4me3 | CD4_ab | 0.000515 | ***1.34E-09*** | -15.7 | 25.7 | ***4.88E-14*** | 3.09E-02 |
| rs138300818 | CCACGTTG-/GCCGGCTGGTCTTGAAC |  | G | state12 | CD3p_CD4p_CD8p | 1.21E-05 | ***3.39E-10*** | 1.8 | 28.1 | ***7.47E-13*** | 1.09E-02 |
| rs138300818 | CACGTTG-/GCCGGCTGGTCTTGAACTC |  | G | state10 | CD3p_CD4p_CD8p | 2.69E-06 | ***1.35E-10*** | 7.6 | 29.7 | ***1.88E-12*** | 1.51E-02 |
| rs138300818 | CAGGAGTTCAAGACCAGCCTGG-/CCAACGTGG |  | G | H3K4me3 | CD8_ab | ***9.23E-11*** | ***8.86E-16*** | 26.2 | 46.4 | ***3.37E-12*** | 1.41E-01 |
| rs138300818 | AGAGATGGGGTTTCACCACGTTG-/GCCGGC |  | G | state9 | CD3p_CD4p_CD8p | 4.08E-06 | ***2.62E-10*** | 1.1 | 27.3 | ***1.98E-10*** | 6.96E-02 |
| rs138300818 | CCAGCCTGG-/CCAACGT |  | G | H3K4me3 | CD4_ab | 0.00151 | ***4.10E-08*** | -4.9 | 21.6 | ***7.95E-10*** | 1.67E-01 |
| rs138300818 | GGTCAGGAGTTCAAGACCAGCCTGG-/CCAAC |  | G | state7 | CD3p_CD4p_CD8p | ***3.14E-14*** | ***8.84E-18*** | 37.6 | 54.1 | ***2.42E-09*** | 2.04E-01 |
| rs138300818 | TCAAGACCAGCCTGG-/CCAACGT |  | G | H3K27me3 | CD3n_CD4p_CD8p | 7.73E-06 | ***7.24E-09*** | -7.6 | 19.0 | ***4.99E-05*** | 5.28E-02 |
| rs138300818 | CCTGG-/CCAACGTGGTGAAACCCCATCTCTAC |  | G | state12 | CD3n_CD4p_CD8p | ***1.64E-08*** | ***4.94E-11*** | 22.4 | 30.3 | ***1.42E-04*** | 4.93E-01 |
| rs113297984 | TGAACTCAGGAGGCAGAG/AGTTGCAGTGAGC | G | A | state12 | CD3p_CD4p_CD8p | ***9.16E-17*** | ***5.74E-13*** | 47.1 | 37.4 | ***8.25E-10*** | 3.07E-01 |
| rs1089652 | C/TTTATTTTTTT | C | T | state12 | CD3n_CD4p_CD8p | 0.000026 | ***4.19E-08*** | 10.3 | 21.5 | ***2.43E-05*** | 3.26E-01 |
| rs1089652 | GAAAAAAATAAG/ATAT | C | T | state12 | CD3n_CD4p_CD8p | 2.88E-05 | ***7.47E-08*** | 9.8 | 20.7 | ***9.44E-05*** | 3.35E-01 |
| rs1089652 | ATAC/TTTATTTTTTTCTCTTAATTTTTGTCT | C | T | state12 | CD4_ab | ***7.91E-10*** | ***1.40E-12*** | 26.7 | 34.3 | ***1.22E-04*** | 5.53E-01 |
| rs1089652 | AGAGAAAAAAATAAG/A | C | T | state12 | CD3p_CD4p_CD8p | 0.000022 | ***7.86E-08*** | 9.5 | 20.7 | ***1.37E-04*** | 2.96E-01 |
| rs1089652 | AAAAATTAAGAGAAAAAAATAAG/ATATCAA | C | T | H3K27ac | CD3p_CD4p_CD8p | ***2.06E-07*** | ***1.10E-09*** | 18.7 | 26.0 | 2.42E-03 | 5.24E-01 |
| rs1089652 | AAAAAAATAAG/A | C | T | H3K27me3 | CD3n_CD4p_CD8p | 8.85E-06 | ***6.68E-08*** | 10.3 | 21.5 | 4.73E-03 | 4.27E-01 |
| rs1089652 | C/TTTATTTTTTTCTCT | C | T | H3K27ac | CD3n_CD4p_CD8p | 2.42E-05 | ***1.61E-07*** | 8.8 | 19.9 | 7.63E-03 | 6.47E-01 |
| rs72973797 | AAATAAAATAAAATAAAG/ATAAATATAAAA | G | A | H3K27ac | CD3n_CD4p_CD8p | ***6.38E-09*** | ***3.03E-12*** | 23.8 | 30.8 | ***3.35E-05*** | 8.04E-01 |
| rs72973797 | AAATAAAATAAAG/ATA | G | A | state12 | CD3n_CD4p_CD8p | 1.36E-05 | ***2.58E-08*** | 11.7 | 21.9 | ***3.92E-05*** | 3.74E-01 |
| rs72973797 | ATATTTAC/TTTTATTTTATTTTATTTCTTGA | G | A | H3K4me3 | CD3n_CD4p_CD8p | ***3.61E-11*** | ***3.42E-14*** | 28.9 | 33.6 | ***4.97E-05*** | 8.53E-01 |
| rs72973797 | AAAATAAAG/ATAAATATAAAAACAAGAAAAG | G | A | H3K4me3 | CD3p_CD4p_CD8p | ***6.97E-10*** | ***1.95E-12*** | 26.9 | 34.9 | 6.80E-04 | 6.37E-01 |
| rs72973797 | AAAG/ATAAATATAAAA | G | A | state12 | CD4_ab | ***2.18E-07*** | ***1.03E-09*** | 19.1 | 24.7 | 1.23E-03 | 6.76E-01 |
| rs72973797 | AAAG/ATAAATATAAAA | G | A | state11 | CD3p_CD4p_CD8p | 2.66E-06 | ***1.17E-08*** | 14.5 | 23.2 | 1.76E-03 | 7.11E-01 |
| rs72973797 | TTTTTATATTTAC/TTTTATTTT | G | A | state11 | CD3p_CD4p_CD8p | ***7.53E-08*** | ***4.48E-10*** | 16.6 | 27.7 | 3.21E-03 | 6.22E-01 |
| rs72973797 | AAATAAAATAAAG/ATAAATATA | G | A | H3K4me3 | CD3n_CD4p_CD8p | ***2.71E-08*** | ***1.97E-10*** | 21.3 | 29.1 | 4.50E-03 | 7.26E-01 |
| rs72973797 | AAAATAAAG/ATAAATA | G | A | H3K27ac | CD3n_CD4p_CD8p | 8.73E-07 | ***4.87E-09*** | 16.5 | 24.6 | 5.66E-03 | 7.62E-01 |
| rs11753289 | TAAACAGATAAAAAATTA/CTAGATATTTAAA | T | G | H3K27ac | CD3n_CD4p_CD8p | ***1.51E-10*** | ***1.34E-07*** | 29.1 | 18.3 | ***7.83E-05*** | 5.07E-01 |
| rs11753289 | AAAAATTA/CTAGATATTTAAAA | T | G | H3K27ac | CD3p_CD4p_CD8p | ***8.53E-08*** | 4.45E-05 | 20.3 | 9.6 | ***2.92E-04*** | 3.77E-01 |
| rs11753289 | ATATTTTCTATCAATTTTTTAAATATCTAT/G | T | G | H3K4me3 | CD4_ab | ***4.76E-11*** | ***1.31E-08*** | 29.4 | 22.8 | 6.53E-04 | 7.20E-01 |
| rs41285280 | AAAATATGCG/AGCAAAGATAAAATGTCTTT | C | T | state12 | CD3n_CD4p_CD8p | 0.000064 | ***4.46E-07*** | 8.6 | 17.9 | 1.16E-03 | 4.15E-01 |
| rs802733 | ATTTTTTATTTGAGTTCAGTATTGGTCG/ATA | G | A | H3K4me3 | CD4_ab | 2.73E-05 | ***1.94E-07*** | 9.1 | 18.8 | 4.39E-03 | 6.19E-01 |
| rs12111314 | TAACATAATAAACATTACC/TGGGTAAAAAA | C | T | H3K27ac | CD4_ab | 2.85E-05 | ***2.04E-07*** | 10.3 | 19.0 | 6.51E-03 | 4.81E-01 |

P_REF_ and P_ALT_: p-value for motif overlap with the DNA sequence containing the REF/ALT alleles. Sc_REF_ and Sc_ALT_: Score value for motif overlap with the DNA sequence containing the REF/ALT alleles, larger values are more significant. E_Diff_ (P_REF_ - P_ALT_): Significance for allelic difference in P-values, calculated based on observed distribution of p-value differences in each of the 17 histone mark – cell type combinations. E_Diff_ (Sc_REF_ - Sc_ALT_): Significance for allelic difference in scores, calculated based on observed distribution of score differences in each of the 17 histone mark – cell type combinations.

## Supplementary **Table S6: DeepBind transcription factor binding motifs that are affected by T1D SNPs that also affect thymocyte motifs.**

|  |  | **SNP** | **motif allele** | **ID** | **Sc_REF_** | **Sc_ALT_** | **Diff** | **Protein** | **Type** | **Species** | **Family** | **Experiment** | **Experiment.Details** |
| --- | --- | --- | --- | --- | --- | --- | --- | --- | --- | --- | --- | --- | --- |
| AAT1D | *PTPRK/ THEMIS* | rs138300818 | ALT | D00072.001 | **30,90** | 0,02 | 30,87 | Rfx7 | TF | *Mus musculus* | RFX | PBM | [DREAM5ID=TF_53, Array=ME] |
| AAT1D | *PTPRK/ THEMIS* | rs138300818 | ALT | D00619.003 | **5,13** | -1,96 | 7,09 | RFX5 | TF | *Homo sapiens* | RFX | SELEX | [CloneType=DBD, Primer=TGGAGC30NGAT, Cycle=4, Batch=AI] |
| AAT1D | *PTPRK/ THEMIS* | rs138300818 | ALT | D00616.002 | 4,07 | -1,44 | 5,51 | RFX3 | TF | *Homo sapiens* | RFX | SELEX | [CloneType=DBD, Primer=TGGCTT20NGA, Cycle=3, Batch=AC] |
| AAT1D | *PTPRK/ THEMIS* | rs138300818 | ALT | D00069.001 | 2,04 | 4,28 | 2,24 | Nr5a2 | TF | *Mus musculus* | Nuclear receptor | PBM | [DREAM5ID=TF_50, Array=ME] |
| AAT1D | *PTPRK/ THEMIS* | rs138300818 | ALT | D00579.002 | 0,98 | 3,07 | 2,09 | PHOX2A | TF | *Homo sapiens* | Homeo-  domain | SELEX | [CloneType=DBD, Primer=TGACTC20NGA, Cycle=3, Batch=Y] |
| T1D | *AFF3* | rs66733041 | REF | D00054.001 | 3,30 | 0,19 | 3,11 | Atf3 | TF | *Mus musculus* | bZIP | PBM | [DREAM5ID=TF_35, Array=ME] |
| T1D | *AFF3* | rs66733041 | REF | D00410.003 | 3,24 | 0,95 | 2,29 | GATA3 | TF | *Homo sapiens* | GATA | SELEX | [CloneType=DBD, Primer=TGTCGT20NGA, Cycle=4, Batch=AC] |
| T1D | 7p15.2 | rs142852921 | REF | D00794.047 | 4,57 | 2,23 | 2,34 | POLR2A | TF | *Homo sapiens* |  | ChIP-seq | [CellLine=MCF-7, Antibody=Pol2, Lab=UT-A] |

motif allele: allele that matches a thymocyte motif. ID: Deepbind transcription factor ID. Sc_REF/ Sc_ALT: Deepbind score for REF/ALT alleles; Diff: difference in REF/ALT scores. Protein: motif binding protein. Type: type of the protein, either transcription factor (TF) or RNA-bindig protein (RBP). Family: TF/RBP superfamily.

## Supplementary Table S7: Transcription factor binding affinity calculated with sTRAP for SNPs that also affect thymocyte motifs

| **Pheno** | **Locus** | **SNP REF ALT** | **Rank** | **Dif log_10_(p)** | **P REF** | **P ALT** | **Matrix_ID** | **Matrix_name** |
| --- | --- | --- | --- | --- | --- | --- | --- | --- |
| AAT1D | *PTPRK/ THEMIS* | rs138300818 REF (–) ALT G | 1 | 2.32 | 0.0025 | 0.52 | PB0055.1 | Rfx4_1 |
| AAT1D | *PTPRK/ THEMIS* | rs138300818 REF (–) ALT G | 2 | 2.24 | 0.0052 | 0.91 | PB0056.1 | Rfxdc2_1 |
| AAT1D | *PTPRK/ THEMIS* | rs138300818 REF (–) ALT G | 3 | 1.81 | 0.011 | 0.70 | PB0054.1 | Rfx3_1 |
| AAT1D | *PTPRK/ THEMIS* | rs138300818 REF (–) ALT G | 4 | 1.69 | 0.013 | 0.63 | PF0034.1 | CYTAGCAAY |
| AAT1D | *PTPRK/ THEMIS* | rs138300818 REF (–) ALT G | 5 | -1.49 | 0.0044 | 0.00014 | PB0131.1 | Gmeb1_2 |
| AAT1D | *PTPRK/ THEMIS* | rs138300818 REF (–) ALT G | 6 | 1.31 | 0.031 | 0.63 | MA0365.1 | RFX1 |
| T1D | *AFF3* | rs66733041 REF CTATGATGATAC ALT (-) | 1 | 2.61 | 0.0018 | 0.73 | PH0161.1 | Six1 |
| T1D | *AFF3* | rs66733041 REF CTATGATGATAC ALT (-) | 2 | 2.23 | 0.0047 | 0.80 | PH0162.1 | Six2 |
| T1D | *AFF3* | rs66733041 REF CTATGATGATAC ALT (-) | 3 | 1.72 | 0.012 | 0.64 | PH0166.1 | Six6_2 |
| T1D | *AFF3* | rs66733041 REF CTATGATGATAC ALT (-) | 4 | 1.63 | 0.017 | 0.74 | PH0163.1 | Six3 |
| T1D | *AFF3* | rs66733041 REF CTATGATGATAC ALT (-) | 5 | 1.39 | 0.037 | 0.89 | PB0038.1 | Jundm2_1 |
| T1D | *AFF3* | rs66733041 REF CTATGATGATAC ALT (-) | 6 | 1.36 | 0.016 | 0.37 | MA0392.1 | STB5 |
| T1D | *AFF3* | rs66733041 REF CTATGATGATAC ALT (-) | 7 | 1.33 | 0.013 | 0.28 | MA0246.1 | so |
| T1D | *AFF3* | rs66733041 REF CTATGATGATAC ALT (-) | 8 | 1.32 | 0.014 | 0.30 | PH0164.1 | Six4 |
| T1D | *AFF3* | rs66733041 REF CTATGATGATAC ALT (-) | 9 | 1.23 | 0.036 | 0.61 | PH0165.1 | Six6_1 |
| T1D | 7p15.2 | rs142852921 REF G ALT (-) | 1 | 1.56 | 0.0026 | 0.10 | CN0019.1 | LM19 |
| T1D | 7p15.2 | rs142852921 REF G ALT (-) | 2 | 1.16 | 0.0168 | 0.24 | CN0134.1 | LM134 |

Dif log10(p): diffenrece in log10(p-values) for REF vs ALT alleles. All transcription factors with | Dif log10(p)| ≥1 and p-value for REF or ALT are shown.

## Supplementary **Table S8. Thymocyte motifs overlapping T1D credible set SNPs with marked difference in motif-sequence similarity between the reference and alternative alleles.** Significant motif overlap was considered as *P*_REF_ or *P*_ALT_ < 0.01/253 thymocyte motifs/2467 autoimmune disease SNPs = 1.60×10^-8^. Significant allelic difference was calculated based on observed distribution of p-value differences, separately for each of the 17 histone mark – cell type combinations and defined as E_Diff_ (P_REF_ - P_ALT_) < 0.01/17 = 5.88×10^-4^. Significant values are indicated with bold.

| **SNP** | **REF** | **ALT** | **LOCUS** | **Matched Seq (REF/ALT)** | **Str** | **P_REF_** | **P_ALT_** | **Sc_REF_** | **Sc_ALT_** | **E_Diff_**  **(P_REF_ - P_ALT_)** | **E_Diff_**  **(Sc_REF_ - Sc_ALT_)** | **hmark** | **ctype** | **Count** |
| --- | --- | --- | --- | --- | --- | --- | --- | --- | --- | --- | --- | --- | --- | --- |
| rs142852921 | **G** |  | 7p15.2 | GTAGTCCCAG/-CTACTTAGGAGGCTGAGG | + | ***4.49E-15*** | 5.04E-05 | 43.5 | -11.6 | ***5.4E-50*** | ***3.6E-07*** | state10 | CD3p_CD4p_CD8p | 12 |
|  |  |  |  | TCAGCCTCCTAAGTAGC/-TGGGACTACAGA | - | ***2.14E-15*** | 7.25E-05 | 44.0 | -8.1 | ***1.52E-43*** | *6.2E-4* | H3K27me3 | CD3n_CD4p_CD8p | * |
| rs66733041 | **CTATGATGATAC** |  | *AFF3* | AGTGCGGTGGTATCATCATAG/-CTCTCTGCA | - | ***1.35E-11*** | 6.72E-03 | 31.3 | -41.2 | ***3.0E-32*** | ***3.8E-04*** | H3K4me3 | CD4_ab | 5 |
| rs71024750 |  | **GTGTGT** | *CENPW* | GTGTGTGTGTGTGTGTGTGTGTGTGTGTGT | + | ***8.43E-12*** | ***1.69E-18*** | 33.4 | 45.7 | ***2.7E-23*** | 4.5E-01 | state9 | CD3p_CD4p_CD8p | 4 |
| rs368755101 | G |  | *AFF3* | TTTC/-TTTTTTTTTTTTTTTTTT | - | 1.11E-07 | ***4.44E-13*** | 20.0 | 30.2 | ***4.3E-16*** | 4.4E-01 | H3K27ac | CD3p_CD4p_CD8p | 6 |
| rs588447 | **A** | C | *PTPN2* | TCTGTTGCCCAGGCTGGAGTGCA/CAT | + | ***2.76E-15*** | ***2.72E-10*** | 39.1 | 28.5 | ***3.6E-15*** | 3.3E-01 | state12 | CD3p_CD4p_CD8p | 8 |
| rs145917030 |  | **GTTGTG** | *AFF3* | CTAAACACACACACACACACAAC/-AC | - | 3.06E-06 | ***4.90E-12*** | 5.0 | 34.3 | ***1.2E-14*** | 6.0E-02 | state12 | CD3n_CD4p_CD8p | 10 |
| rs79092647 | **AAA** |  | *RBM17 IL2RA* | GGGCAAAAGTCTGTCTCAAAAAAAAAAAA/-TGC | + | ***9.47E-14*** | 1.92E-08 | 38.6 | 20.8 | ***1.2E-13*** | 3.2E-01 | state11 | CD3p_CD4p_CD8p | 1 |
| rs7193670 | **T** | A | *DEXI CLEC16A* | GGGATTACAGGCA/TTGAGCCACCGCGCCCG | - | ***1.57E-17*** | ***2.59E-13*** | 48.3 | 38.6 | ***6.8E-13*** | 4.6E-01 | state7 | CD3p_CD4p_CD8p | 9 |
| rs34361002 |  | **AA** | *DEXI CLEC16A* | TAAAAAAAAAAAAAA/- | + | 2.48E-04 | ***7.15E-09*** | 2.1 | 23.8 | ***8.1E-13*** | 4.8E-02 | state12 | CD3p_CD4p_CD8p | 6 |
| rs9401891 | G | **T** | *CENPW* | AG/TATTTTTCTTTTTTTTTTTTT | + | 3.88E-07 | ***1.11E-11*** | 18.4 | 27.4 | ***1.8E-11*** | 4.1E-01 | state10 | CD3p_CD4p_CD8p | 4 |
| rs67878610 | **TTT** |  | *PTPN2* | ATTTATATTAAAATAAAAAA/-T | - | ***7.38E-11*** | 1.87E-06 | 26.4 | 16.0 | ***7.4E-11*** | 3.3E-01 | state10 | CD3p_CD4p_CD8p | 2 |
| rs3862471 | G | **T** | *DEXI CLEC16A* | AAACTCTGTCTCTACTAAAAATACAAAAAT | + | ***2.39E-11*** | ***1.80E-15*** | 31.0 | 39.7 | ***7.9E-11*** | 4.3E-01 | state12 | CD3p_CD4p_CD8p | 4 |
| rs571689 | **C** | T | *FUT2* | CCTGTAATCCCAGCTACTCGGGAGGCTGAG | + | ***1.42E-17*** | ***1.65E-13*** | 49.2 | 38.9 | ***1.5E-10*** | 3.4E-01 | state12 | CD3p_CD4p_CD8p | 4 |
| rs2309755 | G | **A** | *AFF3* | GGGATTACAGGCATGAGCCACCGCACCCA | + | ***1.58E-12*** | ***3.09E-16*** | 35.6 | 46.5 | ***2.1E-10*** | 3.9E-01 | state7 | CD3p_CD4p_CD8p | 6 |
| rs10175599 | C | **T** | *AFF3* | AGACTCTGTCTCAAAAAAAAAAAAAAAAG | - | ***4.75E-10*** | ***2.54E-14*** | 27.6 | 38.4 | ***2.6E-10*** | 3.2E-01 | state10 | CD3p_CD4p_CD8p | 11 |
| rs61634868 | **G** | C | *16q23.1* | GGGATTACAGGCATGCGCCACCACACCCA | - | ***3.98E-16*** | ***1.81E-12*** | 46.3 | 35.4 | ***4.7E-10*** | 4.1E-01 | state7 | CD3p_CD4p_CD8p | 5 |
| rs28665408 | **A** | C | *CD226* | TTATTTTCAATTTTTTTTTTTTTTTTTTGT | - | ***2.93E-13*** | ***1.09E-08*** | 33.7 | 23.1 | ***5.3E-10*** | 6.0E-01 | H3K4me3 | CD4_ab | 9 |
| rs57071364 | **AATAAA** |  | *14q24.1* | CTAATTATCTTTATTTTTATTTTTGTAGA | - | ***1.93E-09*** | 8.72E-05 | 25.3 | 6.5 | ***6.0E-10*** | 1.9E-01 | state12 | CD4_ab | 7 |
| rs6729966 | **A** | C | *AFF3* | AGCCGGGCGCGGTGGCTCACG | + | ***5.51E-12*** | 3.94E-08 | 30.7 | 21.2 | ***1.3E-09*** | 3.8E-01 | state12 | CD3p_CD4p_CD8p | 5 |
| rs147034755 | A | **C** | *FAM98B; SPRED1;RASGRP1* | GGGATTACAGGCATGTGCCACCATGCCCG | - | ***2.64E-12*** | ***8.47E-16*** | 34.7 | 45.6 | ***2.2E-09*** | 3.9E-01 | state7 | CD3p_CD4p_CD8p | 5 |
| rs1790962 | **T** | G | *CD226* | TCTCGCTCTGTCGCCCAGGCTGGAGTGC | + | ***7.86E-16*** | ***2.02E-11*** | 43.2 | 32.1 | ***3.3E-09*** | 5.8E-01 | H3K4me3 | CD3n_CD4p_CD8p | 10 |
| rs1295793 | **C** | G | *14q24.1* | CCTGTAATCCCAGCATTTTGGGAGGCTGAG | - | ***1.05E-16*** | ***5.49E-13*** | 47.3 | 37.2 | ***4.7E-09*** | 3.5E-01 | state12 | CD3p_CD4p_CD8p | 3 |
| rs4851259 | A | **C** | *AFF3* | CTGTAATCCCAGCACTTTGGGAGGCTGAGG | + | ***5.72E-14*** | ***2.02E-18*** | 40.7 | 50.7 | ***6.1E-09*** | 6.4E-01 | H3K27ac | CD3n_CD4p_CD8p | 11 |
| rs1688264 | **T** | G | *FUT2* | AAAAAAAAAAAAAAT | + | ***1.35E-10*** | 2.97E-06 | 25.2 | 15.1 | ***6.6E-09*** | 5.0E-01 | state12 | CD3n_CD4p_CD8p | 6 |
| rs11900482 | G | **C** | *AFF3* | ACACACACATGCACACGCACACACACTCA | + | ***1.09E-09*** | ***2.59E-13*** | 26.4 | 36.3 | ***1.1E-08*** | 3.7E-01 | state12 | CD3p_CD4p_CD8p | 3 |
| rs147955926 | **TT** |  | *14q32.2* | AAAAAAAAAAAAAAA | - | ***3.49E-10*** | 1.31E-06 | 26.9 | 16.0 | ***1.8E-08*** | 3.1E-01 | state12 | CD3p_CD4p_CD8p | 14 |
| rs1989265 | T | **G** | *COBL* | TGAACCTGGGAGGCGAAGGTTGCAGTGAGC | + | ***9.60E-13*** | ***2.98E-16*** | 36.6 | 46.2 | ***3.2E-08*** | 3.8E-01 | state12 | CD3p_CD4p_CD8p | 3 |
| rs3823931 | **T** | C | *7p15.2* | ATTTAAAGTATGTAAAGAAGA | - | ***2.18E-10*** | 2.65E-06 | 25.7 | 15.4 | ***4.8E-08*** | 5.0E-01 | state12 | CD3n_CD4p_CD8p | 1 |
| rs12957037 | **T** | G | *PTPN2* | TTTTTGTATTTTTTGTAGAGACAGAGTTT | + | ***8.19E-17*** | ***1.16E-12*** | 47.3 | 36.2 | ***4.8E-08*** | 4.6E-01 | H3K27me3 | CD3n_CD4p_CD8p | 2 |
| rs7785832 | **T** | C | *7p15.2* | ATTTTTTTAAAAATTATTAGTTAAATTTT | + | ***7.55E-10*** | 3.45E-06 | 25.6 | 14.5 | ***6.2E-08*** | 3.1E-01 | state10 | CD3p_CD4p_CD8p | 1 |
| rs692854 | C | **A** | *FUT2* | TGAACCAGGGAGGCAGAGGTTGCAGTGAGC | + | ***2.36E-12*** | ***1.03E-15*** | 35.2 | 45.0 | ***1.2E-07*** | 3.7E-01 | state12 | CD3p_CD4p_CD8p | 4 |
| rs73067437 | **T** | C | *7p15.2* | TTTGTTTGTTTTTTT | + | ***1.67E-09*** | 6.06E-06 | 24.2 | 13.9 | ***1.3E-07*** | 5.1E-01 | state9 | CD3p_CD4p_CD8p | 4 |
| rs1985869 | **C** | G | *DEXI CLEC16A* | CCTGGCCTCAAGTGATCCTCC | + | ***1.23E-12*** | ***2.51E-09*** | 34.3 | 25.3 | ***1.9E-07*** | 4.1E-01 | state12 | CD3p_CD4p_CD8p | 4 |
| rs111279202 | **T** |  | *DEXI CLEC16A* | TTTTTTTTCTTTTTTTTTTTT | + | ***1.81E-14*** | ***1.67E-10*** | 33.6 | 28.2 | ***1.9E-07*** | 7.1E-01 | H3K27me3 | CD3n_CD4p_CD8p | 4 |
| rs12712070 | **C** | A | *AFF3* | TTGTTGCCCAGGCTGGAGTGGAATGG | - | ***3.13E-13*** | ***9.52E-10*** | 36.9 | 26.4 | ***2.6E-07*** | 3.3E-01 | state10 | CD3p_CD4p_CD8p | 3 |
| rs2452170 | **G** | A | *FUT2* | CCTGTAGTTCCAGCTACTCGGGAGGCTGAG | - | ***2.58E-15*** | ***4.76E-12*** | 44.2 | 33.9 | ***2.7E-07*** | 3.4E-01 | state12 | CD3p_CD4p_CD8p | 2 |
| rs507711 | **C** | T | *FUT2* | CTGACCAACATGGTGAAACTCCATTTCCAC | - | ***7.07E-14*** | ***4.96E-10*** | 36.2 | 27.5 | ***2.8E-07*** | 5.6E-01 | state12 | CD3n_CD4p_CD8p | 1 |
| rs139377078 | AGAG |  | *7p15.2* | ATATTTTTCCATTTGTATGTGTTCTTATTT | - | 1.53E-05 | ***2.04E-09*** | 8.5 | 25.4 | ***2.8E-07*** | 2.5E-01 | state12 | CD4_ab | 1 |
| rs1988588 | **T** | A | *14q32.2* | TGAGACGGAGTCTCGCTCTGTTGCCCAGG | - | ***4.16E-16*** | ***1.18E-12*** | 44.7 | 36.3 | ***2.9E-07*** | 5.9E-01 | state9 | CD3p_CD4p_CD8p | 4 |
| rs507855 | A | **G** | *FUT2* | TCCCAGCTACTCGGGAGGCTGAGGCAGGAG | - | ***4.32E-16*** | ***4.42E-19*** | 46.0 | 57.1 | ***3.0E-07*** | 3.8E-01 | state7 | CD3p_CD4p_CD8p | 1 |
| rs1702877 | **C** | T | *IKZF4 DGKA ERBB3* | TCCCAGCTACTCGGGAGGCTGAGGCAGGAG | - | ***4.42E-19*** | ***4.32E-16*** | 57.1 | 46.0 | ***3.6E-07*** | 4.0E-01 | state7 | CD3p_CD4p_CD8p | 2 |
| rs6715254 | **C** | A | *AFF3* | CTGTAATCCCAGCACTTTGGGAGGCCGAGG | + | ***2.74E-17*** | ***2.06E-13*** | 48.9 | 38.8 | ***3.7E-07*** | 6.2E-01 | H3K27ac | CD3n_CD4p_CD8p | 6 |
| rs13207431 | **C** | T | *CENPW* | TCACGCCATTCTCCTGCCTCAGCCTCCCA | + | ***1.25E-15*** | ***1.14E-12*** | 46.1 | 35.0 | ***4.8E-07*** | 4.0E-01 | state7 | CD3p_CD4p_CD8p | 2 |
| rs60455438 | C | **T** | *FAM98B; SPRED1;*  *RASGRP1* | TCTGTCACCCAGGCTGGAGTGCAGT | + | ***1.46E-09*** | ***9.37E-13*** | 25.6 | 36.1 | ***4.8E-07*** | 3.4E-01 | state12 | CD3p_CD4p_CD8p | 5 |
| rs34813703 | GTG |  | *IKZF4 DGKA ERBB3* | TGACACGCCTGTAATCCCAGTACTTC | - | 1.55E-05 | ***7.08E-09*** | -0.8 | 21.4 | ***4.9E-07*** | 9.0E-02 | H3K27ac | CD3p_CD4p_CD8p | 1 |
| rs113003633 | C | **T** | *7p15.2* | AGTGCAGTGGCATGATCTCGGCTCACTGCA | - | ***2.40E-13*** | ***5.21E-17*** | 38.3 | 49.0 | ***6.0E-07*** | 6.0E-01 | H3K4me3 | CD4_ab | 5 |
| rs1262550 | **A** | C | *CENPW* | AAACAAAAAAAAACA | + | ***3.19E-09*** | 7.20E-06 | 23.2 | 12.3 | ***7.1E-07*** | 3.1E-01 | state10 | CD3p_CD4p_CD8p | 9 |
| rs3781196 | **G** | T | *RNLS* | TCTGTGCCTTGGTTTCCTCACTTGTTAAA | + | ***1.63E-10*** | 2.02E-07 | 28.7 | 18.2 | ***1.1E-06*** | 3.3E-01 | state12 | CD3p_CD4p_CD8p | 1 |
| rs4897180 | **A** | T | *CENPW* | ATCTTGGCTCACTGCAACCTCCGCCTCC | - | ***9.23E-17*** | ***5.62E-13*** | 46.7 | 37.6 | ***1.6E-06*** | 4.9E-01 | H3K27ac | CD4_ab | 4 |
| rs12923098 | **T** | C | *DEXI CLEC16A* | GCCTGGGCAACAGAGCAAGAC | - | ***1.75E-12*** | ***7.63E-09*** | 33.9 | 23.5 | ***2.0E-06*** | 4.9E-01 | H3K4me3 | CD8_ab | 2 |
| rs59254259 | **T** | G | *7p15.2* | ACTGCACTCCAGCCTGGGCGA | + | ***3.00E-13*** | ***6.98E-10*** | 36.9 | 27.2 | ***2.5E-06*** | 5.9E-01 | state11 | CD3p_CD4p_CD8p | 7 |
| rs1985872 | G | **C** | *DEXI CLEC16A* | GCTCACGCCTGTAATTCCAGCACTTT | - | ***5.61E-10*** | ***4.24E-13*** | 26.6 | 37.4 | ***2.6E-06*** | 4.1E-01 | H3K27ac | CD3p_CD4p_CD8p | 5 |
| rs11899489 | **A** | C | *AFF3* | CCTGGGCTCAAGTGATCCTCC | - | ***1.58E-12*** | ***1.51E-09*** | 34.1 | 26.2 | ***2.7E-06*** | 4.7E-01 | state12 | CD3p_CD4p_CD8p | 3 |
| rs4851260 | C | **A** | *AFF3* | GGGATTACAGGCACGTGCCACCATGCCCA | - | ***4.44E-12*** | ***8.72E-15*** | 33.7 | 43.2 | ***3.5E-06*** | 4.5E-01 | state7 | CD3p_CD4p_CD8p | 6 |
| rs507766 | **T** | C | *FUT2* | TAAACATATAAAAAT | - | ***3.14E-09*** | 9.18E-06 | 23.7 | 12.6 | ***3.6E-06*** | 4.6E-01 | state12 | CD3n_CD4p_CD8p | 2 |
| rs7192287 | A | **T** | *DEXI CLEC16A* | GGGATTACAGGCGCCTGCCACCATGCCTG | + | ***1.62E-11*** | ***3.23E-14*** | 31.2 | 41.6 | ***3.6E-06*** | 4.1E-01 | state7 | CD3p_CD4p_CD8p | 4 |
| rs10227673 | G | **A** | *7p15.2* | ATTTTTATTATTTTT | - | 2.12E-06 | ***4.80E-10*** | 15.4 | 25.6 | ***4.3E-06*** | 4.6E-01 | H3K27ac | CD4_ab | 3 |
| rs2548459 | **T** | C | *FUT2* | AACTCCTGACCTCAGGTGATC | - | ***2.16E-13*** | ***5.59E-10*** | 37.2 | 27.5 | ***4.7E-06*** | 6.3E-01 | H3K4me3 | CD3n_CD4p_CD8p | 6 |
| rs28894750 | A | **T** | *FUT2* | AAAAAAAATTAGCCAGGTGTG | - | 3.94E-08 | ***2.26E-11*** | 20.6 | 31.4 | ***5.8E-06*** | 5.6E-01 | state11 | CD3p_CD4p_CD8p | 1 |
| rs506897 | **G** | C | *FUT2* | CCTGTAATCCCAGCACTTTGGGAGGCCGA | - | ***2.34E-17*** | ***5.58E-14*** | 50.5 | 40.6 | ***5.9E-06*** | 6.2E-01 | H3K4me3 | CD3n_CD4p_CD8p | 1 |
| rs6564237 | C | **G** | *16q23.1* | AGGAAGATCGCTTGAGCCCAG | - | 3.52E-08 | ***3.14E-11*** | 21.3 | 30.6 | ***6.3E-06*** | 3.9E-01 | state10 | CD3p_CD4p_CD8p | 2 |
| rs28816386 | A | **G** | *7p15.2* | GGCCTCCCAAAGTGCTGGGATTACAGGCG | - | ***3.18E-14*** | ***1.33E-17*** | 41.7 | 50.1 | ***7.7E-06*** | 5.7E-01 | state12 | CD4_ab | 2 |
| rs3030572 |  | **A** | *DEXI CLEC16A* | AGCTAATTTTTTTTTTAATTAAAATTATCT | + | 5.92E-08 | ***2.56E-11*** | 20.0 | 31.1 | ***8.4E-06*** | 4.5E-01 | state12 | CD4_ab | 2 |
| rs4930045 | **T** | A | *INS* | CCATCCTGGCTAACACAGTGAAACCCCGTC | - | ***9.75E-13*** | ***1.85E-09*** | 35.8 | 25.1 | ***8.5E-06*** | 6.0E-01 | H3K4me3 | CD4_ab | 2 |
| rs75169667 | **A** | G | *CENPW* | AAGAAGGAAAGAGAG | + | ***1.33E-08*** | 2.76E-05 | 21.6 | 10.4 | ***9.2E-06*** | 4.6E-01 | state12 | CD3n_CD4p_CD8p | 1 |
| rs9388496 | **A** | G | *CENPW* | TTATTTAAAAATTTT | - | ***3.67E-09*** | 9.15E-06 | 23.0 | 12.9 | ***9.2E-06*** | 5.0E-01 | H3K4me3 | CD8_ab | 1 |
| rs3850234 | **T** | A | *CENPW* | GTAATCCTAGCAATTTGGGAGGATGAGG | - | ***3.83E-14*** | ***3.77E-11*** | 41.1 | 30.1 | ***9.6E-06*** | 3.1E-01 | state10 | CD3p_CD4p_CD8p | 1 |
| rs71136618 |  | **T** | *DEXI CLEC16A* | TTTTTTTTTTCTTTTTTTTTT | + | ***7.80E-09*** | ***3.37E-12*** | 23.8 | 31.2 | ***1.0E-05*** | 6.4E-01 | H3K27me3 | CD3n_CD4p_CD8p | 4 |
| rs1788100 | **A** | G | *CD226* | AAAAAAAAAAAATAT | + | ***1.90E-09*** | 3.61E-06 | 24.0 | 14.7 | ***1.2E-05*** | 5.3E-01 | state12 | CD3n_CD4p_CD8p | 4 |
| rs2214494 | G | **T** | *COBL* | ATTTTTCCTTTTCACTTAATATTTTTAAAT | + | 6.69E-07 | ***4.25E-10*** | 16.7 | 27.1 | ***1.3E-05*** | 6.1E-01 | H3K4me3 | CD4_ab | 1 |
| rs570794 | T | **C** | *FUT2* | TGAGATGGAGTCTTGCTGTGTCACCCAGG | - | ***3.70E-11*** | ***4.42E-14*** | 30.9 | 40.4 | ***1.4E-05*** | 5.6E-01 | state9 | CD3p_CD4p_CD8p | 3 |
| rs3861458 | G | **A** | *CENPW* | TTTAAATTTAAGTTTACATAATGGATTAT | - | 2.32E-06 | ***1.27E-09*** | 15.3 | 25.1 | ***1.4E-05*** | 5.4E-01 | state12 | CD3n_CD4p_CD8p | 1 |
| rs111869668 | **C** | G | *16q23.1* | CTGGAGTGCAGTGGTGCAATCTTGGCTCC | - | ***2.37E-14*** | ***1.75E-11*** | 41.5 | 31.8 | ***2.1E-05*** | 5.3E-01 | state9 | CD3p_CD4p_CD8p | 4 |
| rs8054218 | **A** | G | *16q23.1* | GCCTGGGTGACAGAGTGAGAC | + | ***1.43E-11*** | 2.39E-08 | 32.1 | 21.5 | ***2.6E-05*** | 4.8E-01 | H3K4me3 | CD8_ab | 2 |
| rs145665108 | TGAAAT |  | *7p15.2* | AAATAAATGAAATTTTAAAAAAATACAAAA | + | 7.23E-08 | ***7.79E-11*** | 18.0 | 30.2 | ***3.0E-05*** | 4.5E-01 | H3K4me3 | CD3p_CD4p_CD8p | 4 |
| rs5815611 | **T** |  | *DEXI CLEC16A* | TTTCTTTTTTTTTTT | + | ***7.12E-10*** | 8.41E-07 | 25.7 | 16.9 | ***3.8E-05*** | 6.6E-01 | H3K4me3 | CD3n_CD4p_CD8p | 1 |
| rs112166936 | A | **C** | *CENPW* | CCTCCCTTCCTCTCTCCCTCC | + | 9.09E-08 | ***7.71E-11*** | 20.4 | 28.5 | ***3.8E-05*** | 7.0E-01 | H3K4me3 | CD3n_CD4p_CD8p | 1 |
| rs28362844 | C |  | *FUT2* | ATGTTTTCTTTTCTTTTTCTTTTCTTTTT | + | 6.06E-06 | ***1.06E-08*** | 13.4 | 22.9 | ***4.5E-05*** | 3.9E-01 | state10 | CD3p_CD4p_CD8p | 1 |
| rs1967332 | **C** | A | *7p15.2* | CCACTGCACCCCAGCCTGGGTGACAAAG | + | ***9.35E-15*** | ***4.60E-12*** | 43.2 | 33.3 | ***4.9E-05*** | 4.4E-01 | H3K27ac | CD3p_CD4p_CD8p | 2 |
| rs9952753 | A | **C** | *PTPN2* | GGGATTACAGGCGTAAACCACCCCGCCCA | + | ***7.74E-11*** | ***3.33E-13*** | 27.8 | 38.2 | ***4.9E-05*** | 4.1E-01 | state7 | CD3p_CD4p_CD8p | 1 |
| rs10758593 | G | **A** | *GLIS3* | AAAAAGATTTTTAAGAAAAACATAAATAA | + | 2.23E-06 | ***2.01E-09*** | 15.3 | 24.6 | ***5.1E-05*** | 5.6E-01 | state12 | CD3n_CD4p_CD8p | 1 |
| rs35536837 |  | **T** | *CENPW* | AAAAAAAAAGGAAAAGAAAAATTATATCA | - | 6.89E-06 | ***9.17E-09*** | 12.6 | 23.2 | ***5.8E-05*** | 5.7E-01 | state11 | CD3p_CD4p_CD8p | 1 |
| rs8180649 | T | **C** | *CENPW* | GAACTCCTGACCTCAGGTGAT | + | ***1.11E-09*** | ***9.33E-13*** | 26.6 | 35.4 | ***6.0E-05*** | 6.8E-01 | H3K27ac | CD3n_CD4p_CD8p | 2 |
| rs12592898 | A | **G** | *CTSH* | AACTCCCGACCTCAGGTGATC | + | ***1.74E-09*** | ***1.83E-12*** | 25.6 | 34.9 | ***6.5E-05*** | 6.5E-01 | H3K4me3 | CD3n_CD4p_CD8p | 1 |
| rs139078271 |  | **G** | *CENPW* | TATAATTTCTGTTCTTTTACATTTGCTGA | - | 5.19E-07 | ***5.28E-10*** | 17.1 | 26.9 | ***7.4E-05*** | 5.1E-01 | state12 | CD4_ab | 1 |
| rs112164743 | **A** | G | *7p15.2* | AGAACTTAAAGTATACTTAAAAAAAAAAA | + | ***5.15E-09*** | 6.73E-06 | 23.7 | 13.2 | ***7.9E-05*** | 4.3E-01 | H3K27ac | CD4_ab | 1 |
| rs67518044 | **TAT** |  | *PTPN2* | AAACAAAACAAATAATACCACAATAAAAA | - | ***7.19E-10*** | 6.70E-07 | 26.9 | 15.0 | ***1.1E-04*** | 4.3E-01 | H3K4me3 | CD8_ab | 1 |
| rs9939397 | **A** | G | *DEXI CLEC16A* | ATATATTATAAATTTTTTTAAAGACAAGG | + | ***3.48E-09*** | 2.80E-06 | 24.5 | 14.0 | ***1.1E-04*** | 4.6E-01 | state12 | CD4_ab | 1 |
| rs1704773 | **A** | G | *FUT2* | ACTCTTAAGAATTTTTTTTTTTTTTTTGA | - | ***9.82E-09*** | 2.78E-06 | 23.0 | 12.4 | ***1.1E-04*** | 3.3E-01 | state12 | CD3p_CD4p_CD8p | 2 |
| rs2847291 | **G** | A | *PTPN2* | GGAGGCGGAGGTTGC | + | ***1.84E-09*** | 1.66E-06 | 26.2 | 15.4 | ***1.1E-04*** | 4.8E-01 | H3K4me3 | CD8_ab | 1 |
| rs184458383 | **C** | T | *16q23.1* | TTAGATTCGGCTGGGTGTGGTGGCTCACGC | + | ***3.37E-11*** | 2.06E-08 | 31.5 | 20.7 | ***1.6E-04*** | 6.0E-01 | H3K4me3 | CD4_ab | 1 |
| rs377093688 | **C** | G | *FAM98B SPRED1 RASGRP1* | GCGATCTCGGCTCACTGCAA | + | ***1.30E-12*** | ***8.49E-10*** | 36.5 | 26.6 | ***1.6E-04*** | 6.2E-01 | H3K4me3 | CD3n_CD4p_CD8p | 1 |
| rs8056098 | G | **A** | *DEXI CLEC16A* | TGGCAATTAGTTTTTTTTTTTTTTTTTTGA | - | 3.47E-07 | ***4.98E-10*** | 16.8 | 27.3 | ***1.7E-04*** | 4.8E-01 | state12 | CD4_ab | 1 |
| rs7184615 | G | **C** | *DEXI CLEC16A* | CCTGGGCTCAAGTGATCATCC | + | ***1.25E-08*** | ***5.32E-11*** | 22.0 | 30.9 | ***1.8E-04*** | 4.1E-01 | state12 | CD3p_CD4p_CD8p | 3 |
| rs141552689 |  | **AA** | *7p15.2* | ATTTTTTTTTTAAATTAAAACAAGTTTT | - | 7.26E-07 | ***1.01E-09*** | 10.8 | 25.9 | ***1.9E-04*** | 3.2E-01 | H3K4me3 | CD8_ab | 1 |
| rs9926367 | **T** | C | *DEXI CLEC16A* | AAGTGTGCTTGTGTGGGTGTG | + | ***1.37E-08*** | 7.99E-06 | 22.8 | 12.0 | ***2.1E-04*** | 5.9E-01 | H3K4me3 | CD3n_CD4p_CD8p | 1 |
| rs75407602 | C | **T** | *TYK2* | TTTGATTTTTTTTTTTTTTCCAGAGATGG | + | 2.18E-06 | ***3.43E-09*** | 14.5 | 24.6 | ***2.1E-04*** | 4.9E-01 | state12 | CD4_ab | 1 |
| rs34752850 | **A** |  | *DEXI CLEC16A* | ATCTTCTTTTTTTTTAAGAAACAAGGTCT | - | ***4.10E-09*** | 2.55E-06 | 20.5 | 2.1 | ***2.4E-04*** | 2.2E-01 | H3K27me3 | CD3n_CD4p_CD8p | 1 |
| rs12416116 | C | **A** | *RNLS* | ATACCTCATTAGAACAACAAAAAAAAAAT | + | 9.55E-07 | ***3.25E-09*** | 13.8 | 24.7 | ***2.6E-04*** | 3.2E-01 | state10 | CD3p_CD4p_CD8p | 1 |
| rs5823237 | T |  | *PTPN2* | ATCGTAGGTCACTGCAGACTCAAACTCC | - | 9.55E-07 | ***1.24E-09*** | 1.3 | 21.5 | ***2.8E-04*** | 1.4E-01 | H3K27ac | CD4_ab | 2 |
| rs2847292 | **G** | T | *PTPN2* | ATGGGGTTTCACCATGTTGGCCAGGCTGGT | + | ***6.79E-15*** | ***4.89E-12*** | 43.1 | 34.0 | ***2.9E-04*** | 4.9E-01 | H3K27ac | CD4_ab | 1 |
| rs28404962 | **T** | C | *7p15.2* | AAAAGTTGAATATTAAATCATAAAAATAA | + | ***1.13E-08*** | 7.50E-06 | 22.7 | 13.2 | ***3.5E-04*** | 4.7E-01 | H3K27ac | CD4_ab | 1 |
| rs12722552 | **C** | T | *RBM17 IL2RA* | TGGTCTCGAACTCCTGACCTCAGGTGATC | + | ***3.54E-18*** | ***1.65E-15*** | 54.0 | 46.1 | ***3.6E-04*** | 6.0E-01 | state12 | CD3n_CD4p_CD8p | 1 |
| rs2847273 | **A** | C | *PTPN2* | TTTTTGTATTTTTAGTAGAGATGGAGTTG | - | ***8.84E-13*** | ***4.46E-10*** | 36.6 | 25.5 | ***3.8E-04*** | 4.6E-01 | H3K27me3 | CD3n_CD4p_CD8p | 1 |
| rs443081 | **C** | T | *19q13.32* | TCCCAGCTACTTGGGAGGCTGAGGTAGGAG | - | ***1.84E-17*** | ***2.23E-15*** | 53.5 | 42.4 | ***4.0E-04*** | 4.0E-01 | state7 | CD3p_CD4p_CD8p | 1 |
| rs1120786 | **T** | G | *CENPW* | TTGTTTTATTGTAAATCGTTATTTTATTAT | + | ***1.68E-09*** | 6.65E-07 | 25.5 | 16.7 | ***4.2E-04*** | 6.7E-01 | H3K4me3 | CD4_ab | 1 |
| rs2292759 | A | **G** | *PTPN2* | CCCGCCCCGCCGCCGACTCCGCGCCGCGC | - | 1.58E-06 | ***9.26E-09*** | 14.5 | 23.4 | ***4.3E-04*** | 4.2E-01 | state12 | CD3p_CD4p_CD8p | 1 |
| rs194739 | **C** | T | *14q24.1* | TCCCAGCTACTCGGGAGGCTGAGGCAGGGG | - | ***3.64E-17*** | ***4.03E-15*** | 52.1 | 41.0 | ***5.2E-04*** | 4.0E-01 | state7 | CD3p_CD4p_CD8p | 2 |
| rs200395517 |  | **T** | *19q13.32* | ATTTTTTTTTTCTTTTTCTTTTTATTGTTT | + | 1.71E-08 | ***4.86E-11*** | 22.4 | 29.3 | ***5.2E-04*** | 7.3E-01 | H3K4me3 | CD4_ab | 1 |
| rs11680485 | **A** | G | *AFF3* | TCTAAAAAATAAAATAAAATA | + | ***5.74E-10*** | 2.16E-07 | 27.7 | 16.6 | ***5.5E-04*** | 5.8E-01 | H3K4me3 | CD3n_CD4p_CD8p | 1 |

Count: number of histone modification/ state – cell type combinations where there was significant allelic difference (p<5.88×10^-4^), out of maximum 17. *For rs142852921, in addition to the pseudo-chromatin state 10, also results for the histone modification peak with strongest allelic difference are given.

Supplementary Figure S1: THEMIS and PTPRK expression in single cell RNA sequencing of developing human immune system in thymus**.** Data from *Suo et al, Science 2022*, accessed through <https://developmentcellatlas.cellgeni.sanger.ac.uk/fetal-immune/pfi/>

Early double negative T cells (DN(early)_T)


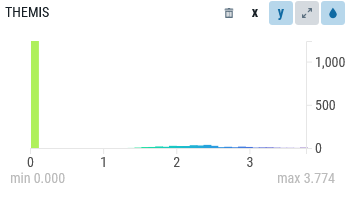

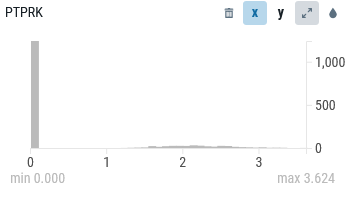

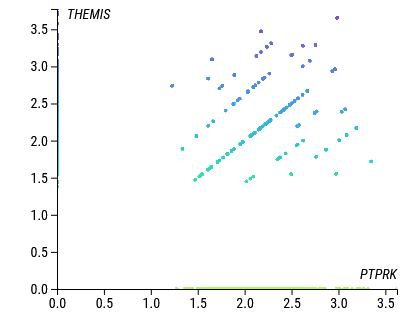


Double negative (P) T cells (DN(P)_T)


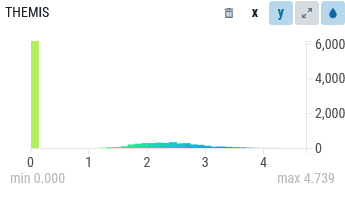

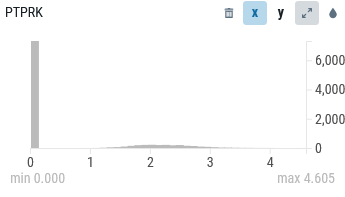

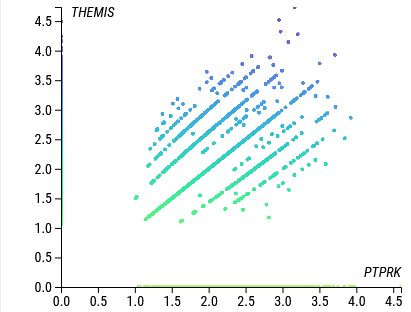


Double negative (Q) T cells (DN(Q)_T)


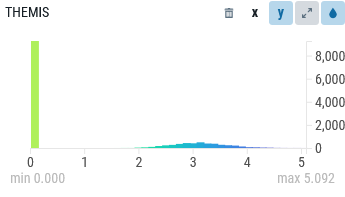

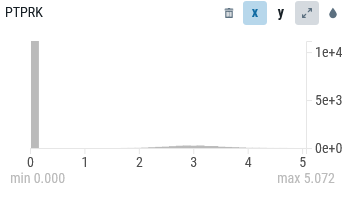

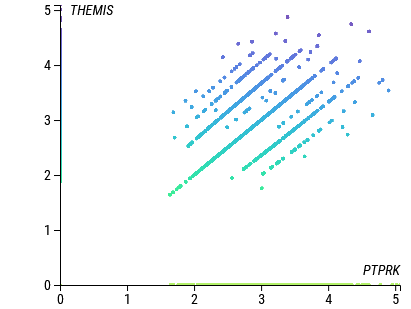


Double positive (P) T cells (DP(P)_T)


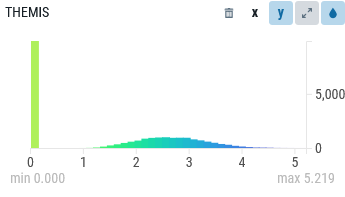

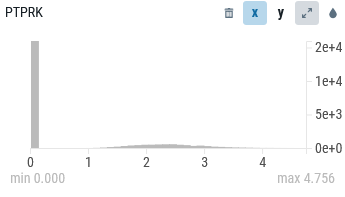

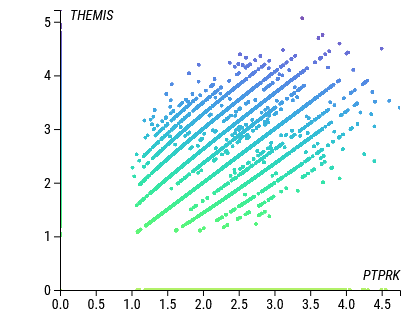


Double positive (Q) T cells DP(Q)_T


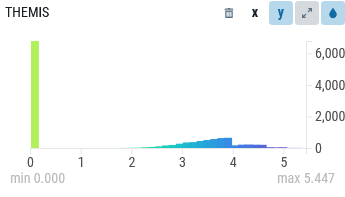

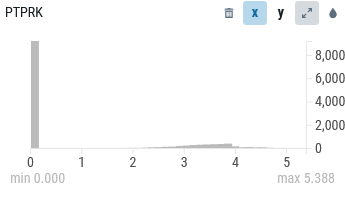

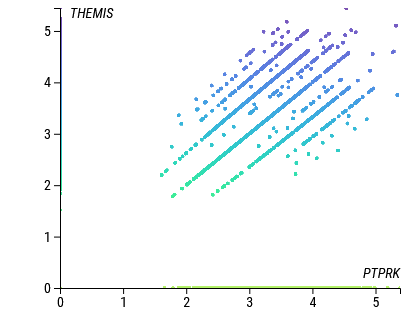


**Supplementary Figure S1, continued**

αβ entry T cells (ABT(Entry))


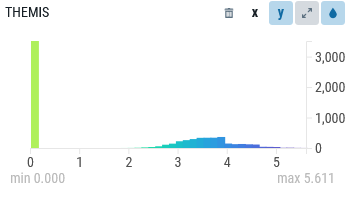

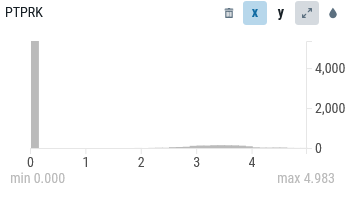

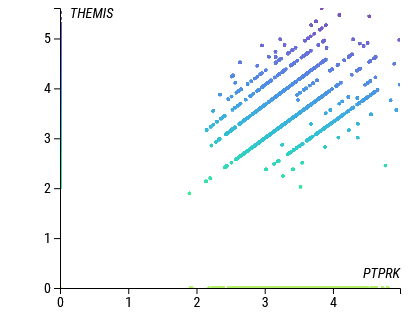


CD4^+^ T cells


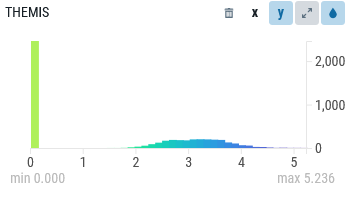

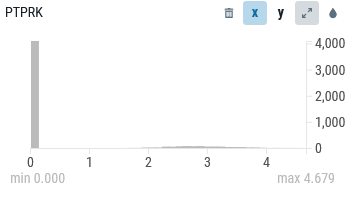

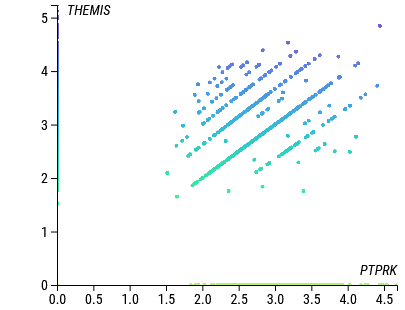


CD8^+^ T cells


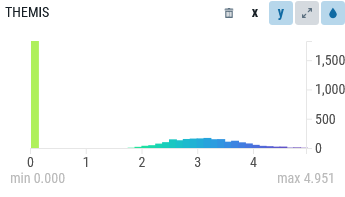

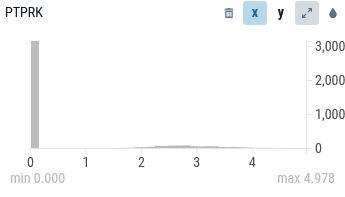

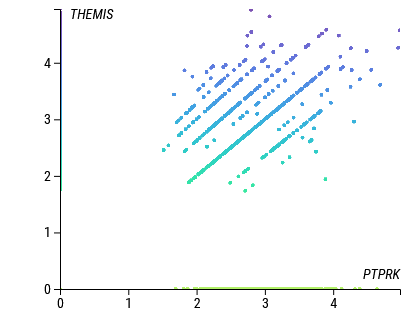


CD8AA T cells


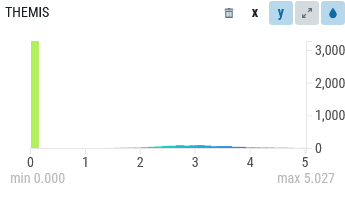

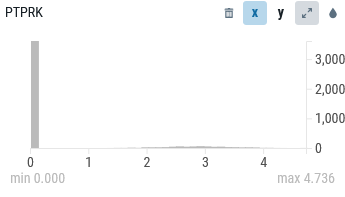

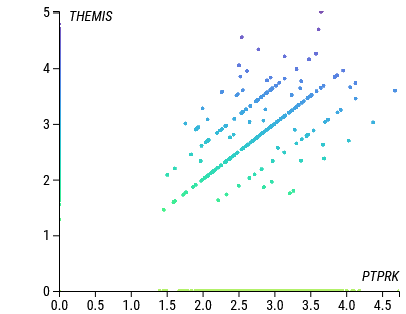


T regulatory cells (TREG)


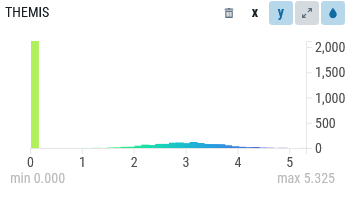

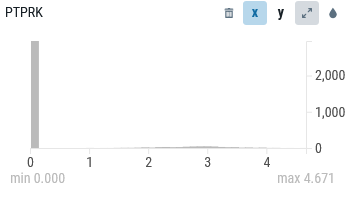

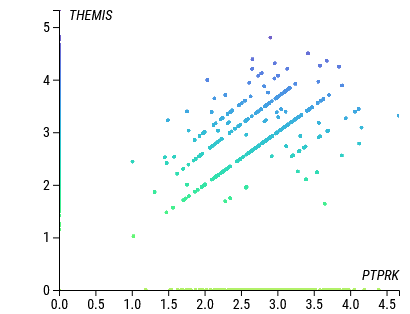


**Supplementary Figure S1, continued**

Type 1 innate T cells (TYPE_1_INNATE_T)


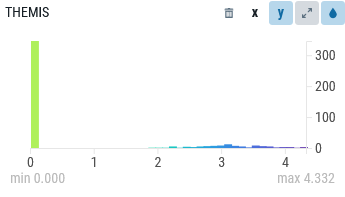

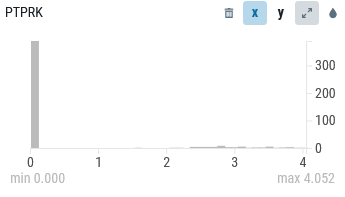

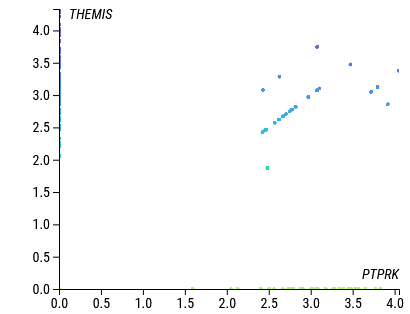


Type 3 innate T cells (TYPE_3_INNATE_T)


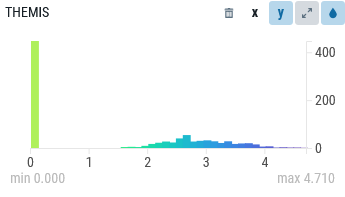

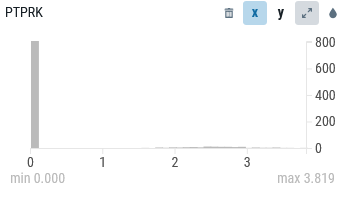

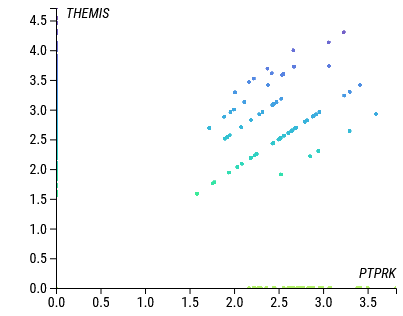


Cycling T cells (CYCLING_T)


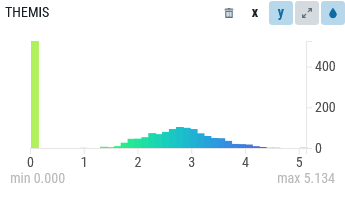

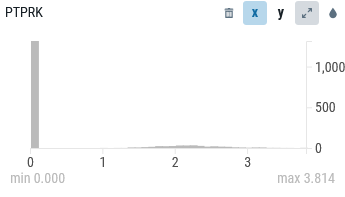

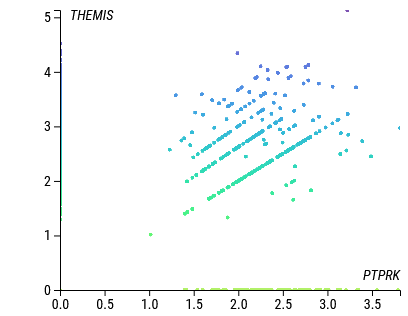


**Supplementary Figure S1, continued**

Thymus, all above (Early double negative[DN], P/Q DN, P/Q double positive [DP], αβ entry, CD4+, CD8+, CD8AA, Treg, type 1/3 innate, and cycling T cells)


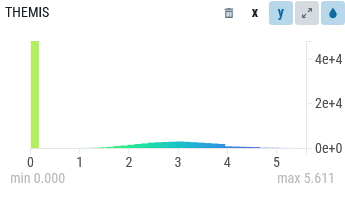

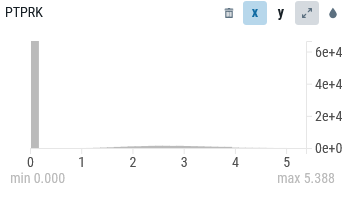


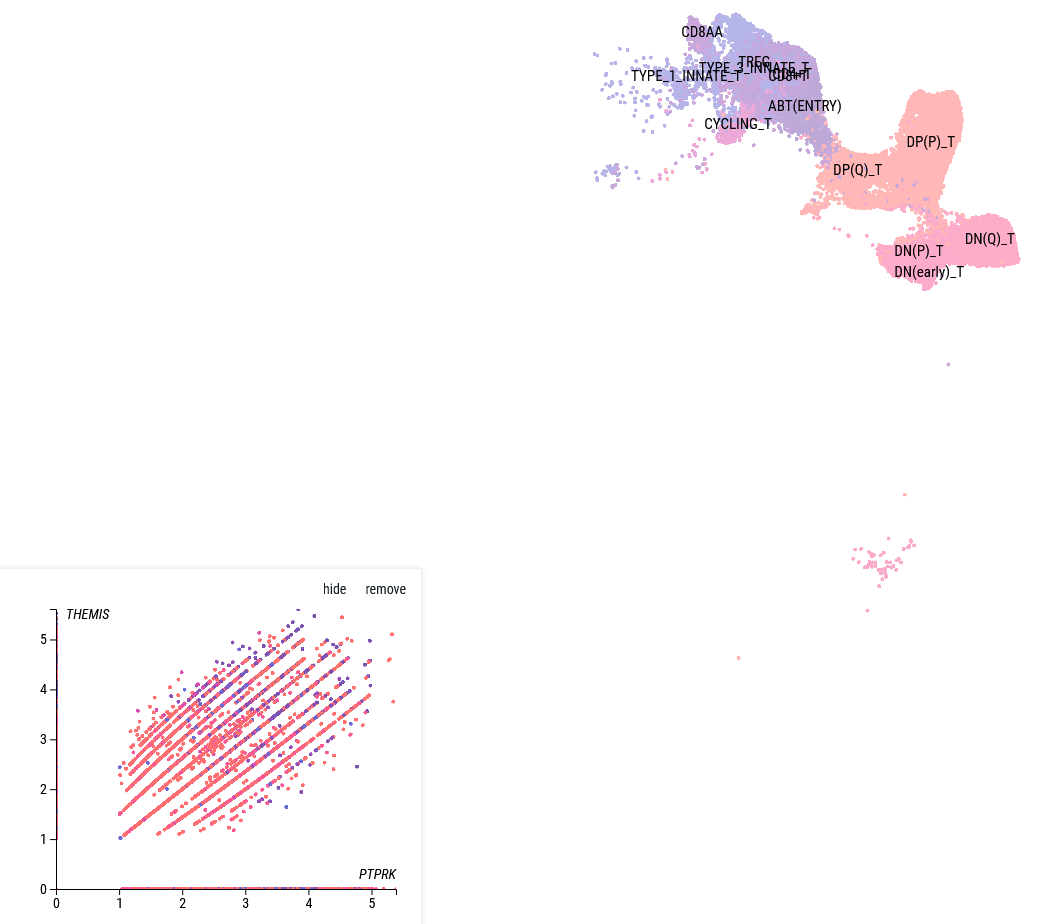


**Supplementary Figure S1, continued**

Thymus, all other (excluding low quality or high mitochondrial cells)


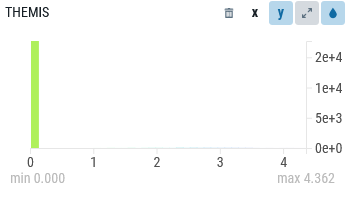

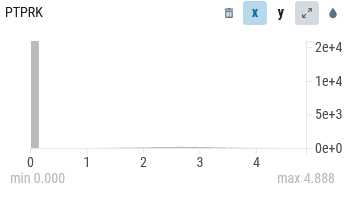


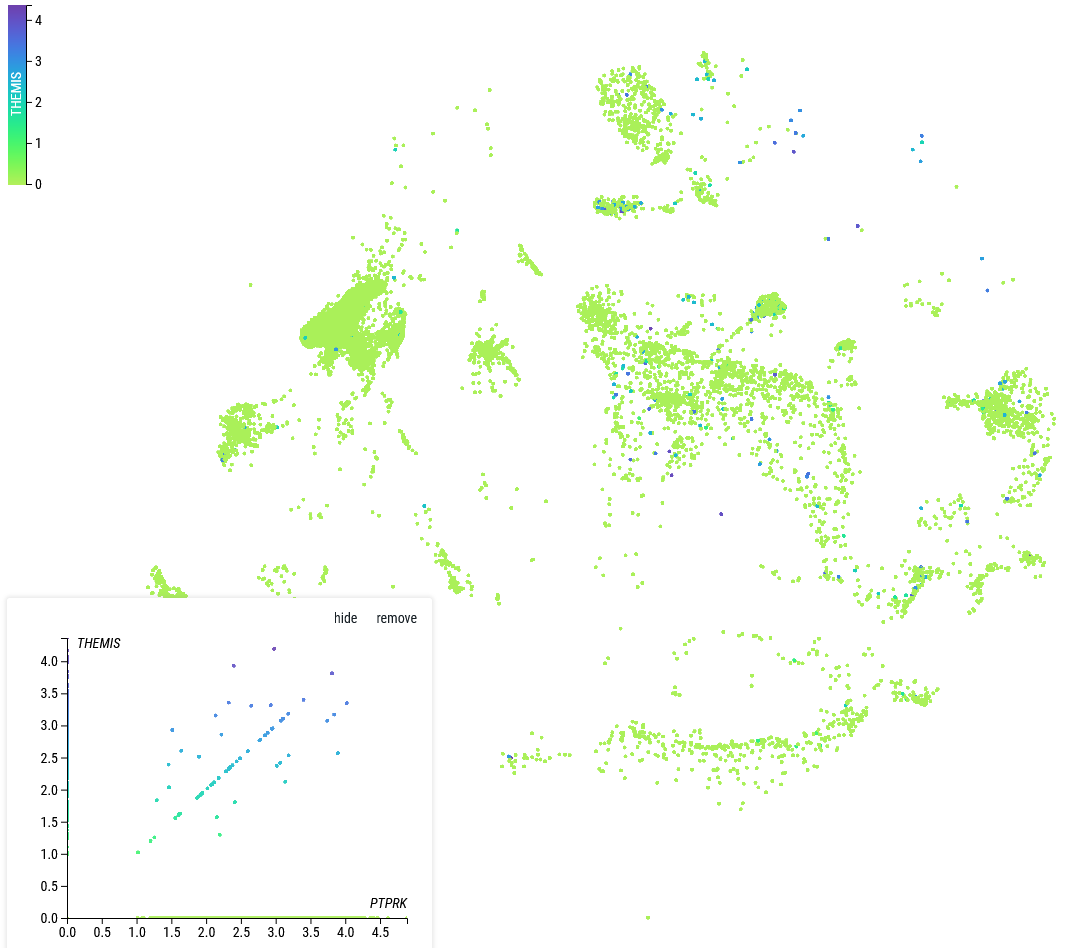


**Supplementary Figure S1, continued**

Thymus, all


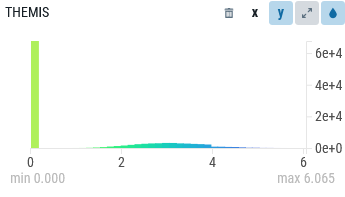

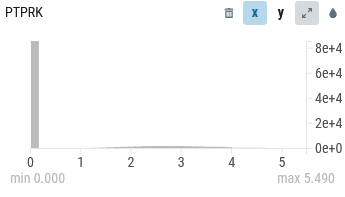


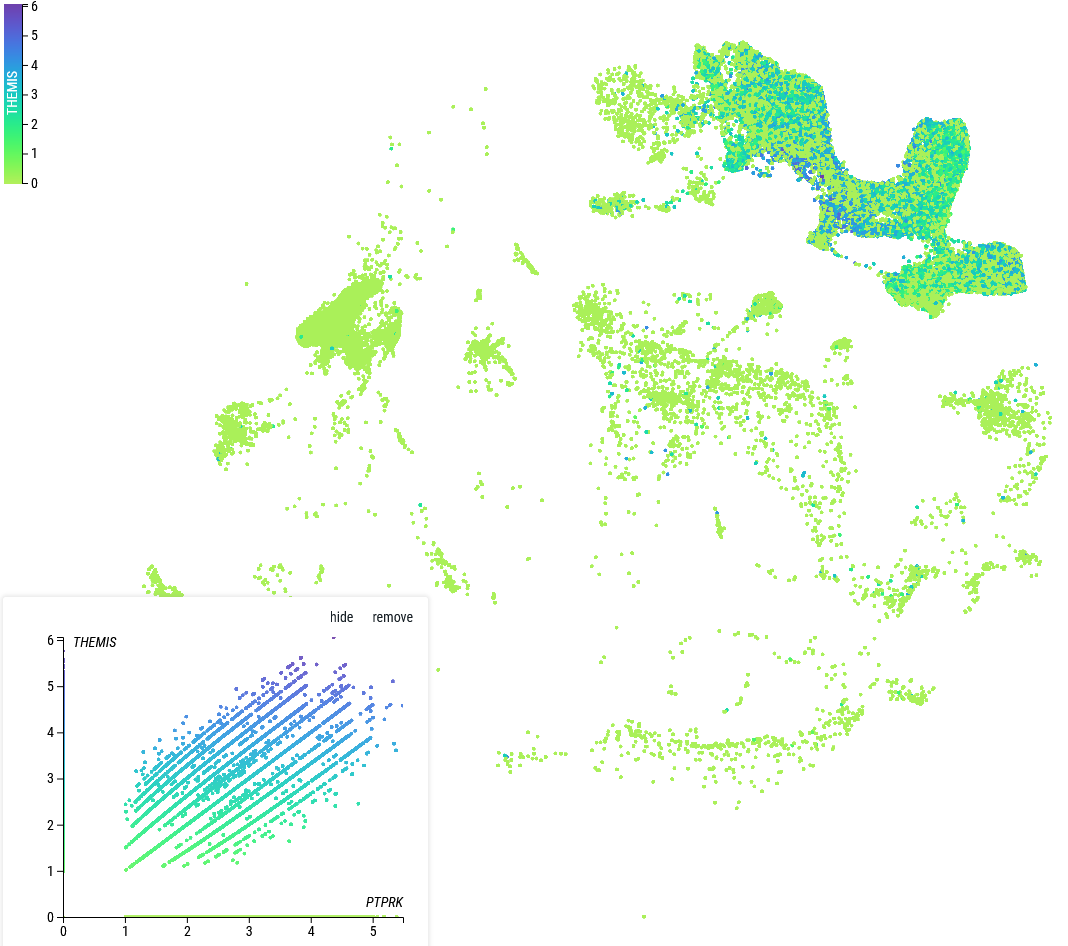


## Supplementary Figure S2. Overlap between the discovered thymocyte motifs and known transcription factor (TF) binding motifs. For each TF, only the thymocyte motif from the cell type and histone modification peak combination with the lowest q-value is shown.

**A: ZNF263, H3K4me3: CD3- CD4+ CD8+ q=1.35×10^-7^ B: SP2, H3K4me3: CD3- CD4+ CD8+ q=1.32×10^-6^**


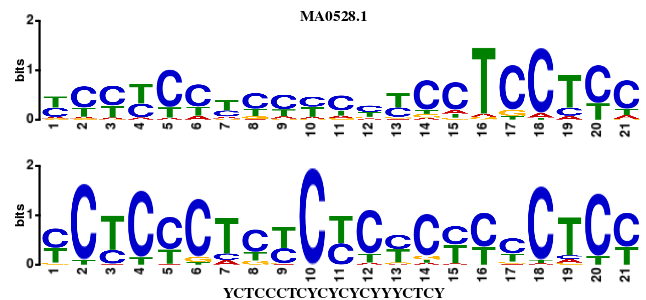

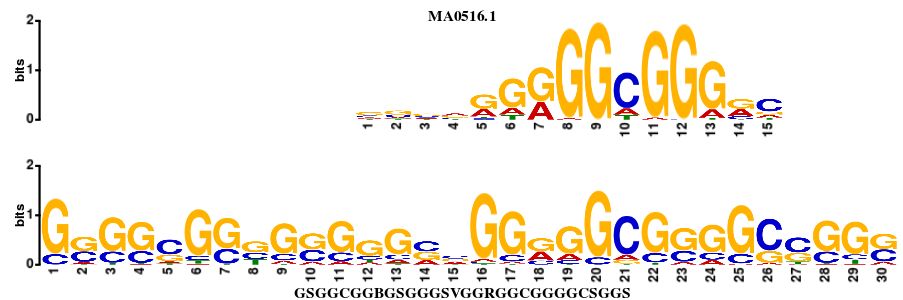


**C: SP1, H3K4me3: CD3- CD4+ CD8+ q=7.19×10^-5^ D: Zfp281 (Znf281), H3K27ac: CD3+ CD4+ CD8+ q=3.75×10^-4^**


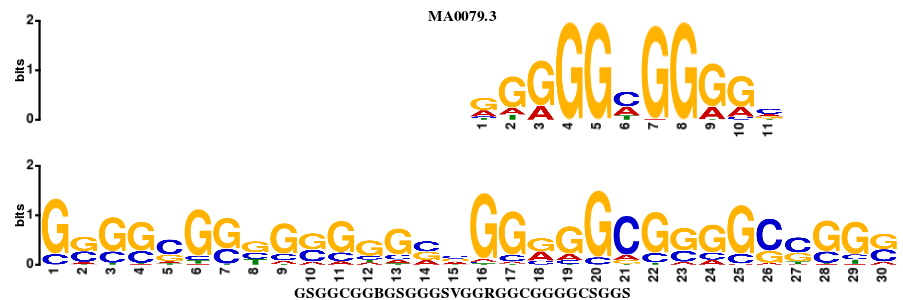

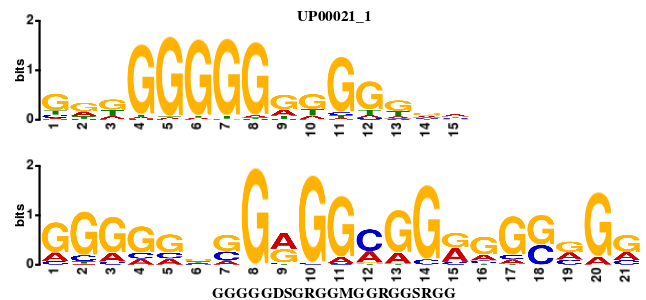


**E: SP4, H3K4me3: CD3- CD4+ CD8+ *q*=5.36×10^-4^ F: KLF16, H3K4me3: CD8+ αß *q*=9.77×10^-4^**


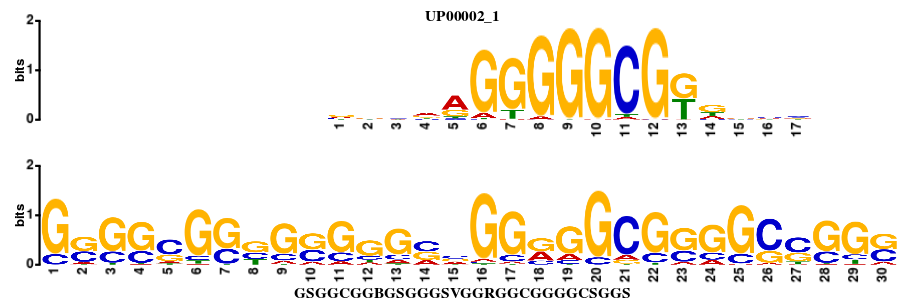

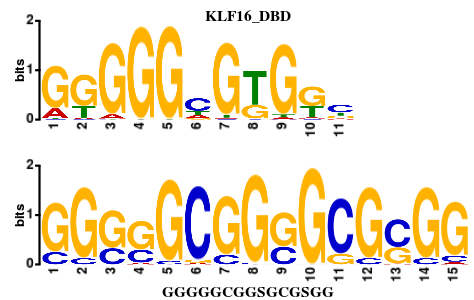


**G: KLF5, H3K4me3: CD8+ αß *q*=2.43×10^-3^ H: SP3, H3K4me3: CD8+ αß *q*=3.04×10^-3^**


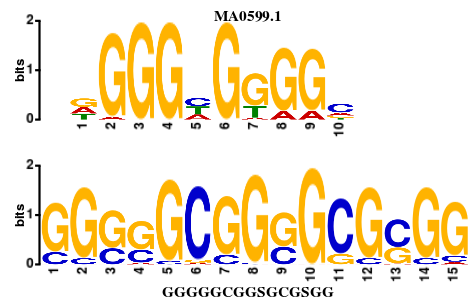

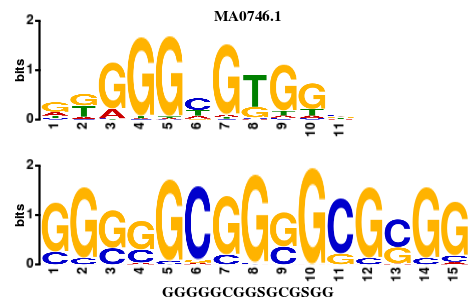


**I: RREB1, Stete12: CD3- CD4+ CD8+ *q*=3.85×10^-3^ J: Zfx, H3K4me3: CD8+ αß *q*=4.17×10^-3^**


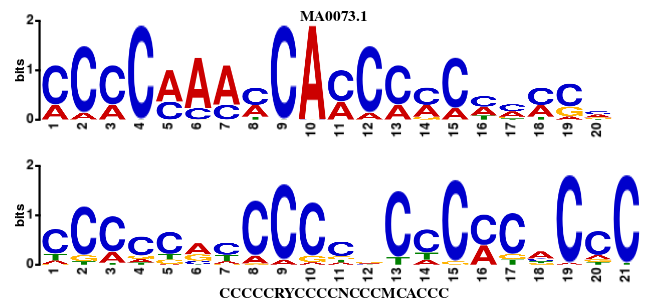

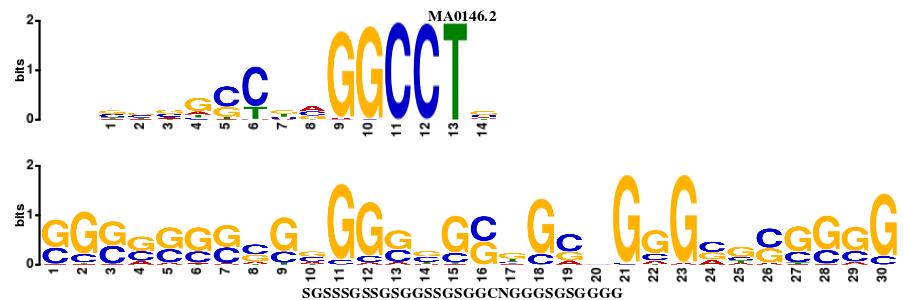


**K: Zfp740 (Znf740), H3K4me3: CD8+ αß *q*=9.11×10^-3^**


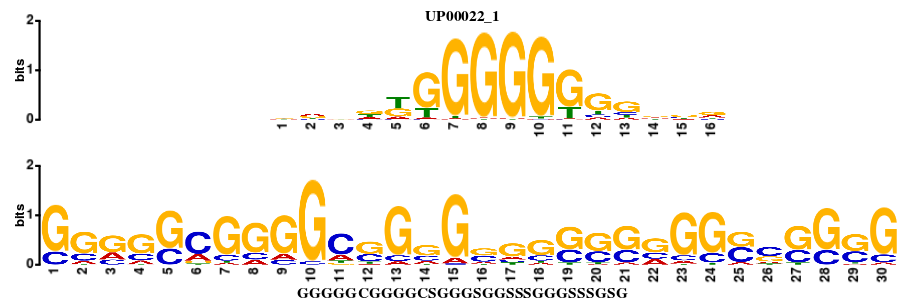


## Supplementary Figure S3. Predicted TF binding for all SNPs in credible set for age at diabetes diagnosis based on deepbind neural network predictions. A: X-axis reports binding for REF allele, y-axis for the ALT allelle. SNP – TF pairs with largest difference between alleles are annotated. B: Table of all SNP - TF pairs where allelic difference was larger than 5 standard deviations (≥6.43) of difference distribution at least with one TF binding; differences ≥6.43 are highlighted with bold.

**A**


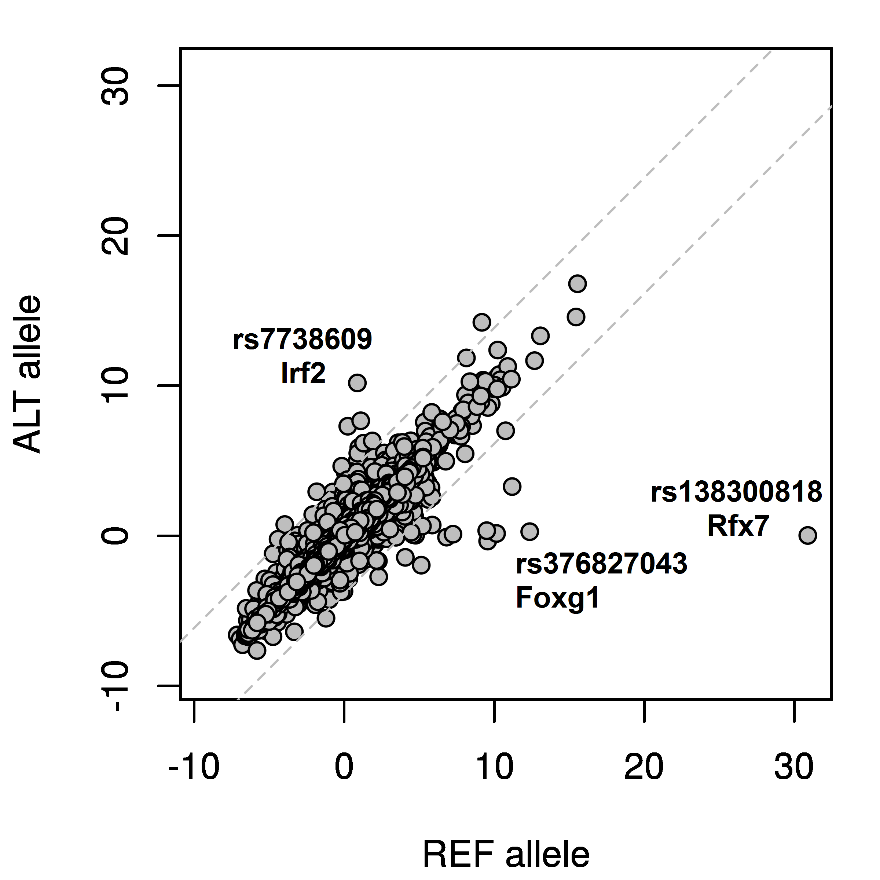


**B**

|  |  | **rs13204742** | | **rs802747** | | **rs376827043** | | **rs1089652** | | **rs7738609** | | **rs138300818** | |
| --- | --- | --- | --- | --- | --- | --- | --- | --- | --- | --- | --- | --- | --- |
| **TF ID** | **TF name** | **REF G** | **ALT T** | **REF G** | **ALT A** | **REF GTTT** | **ALT -** | **REF C** | **ALT T** | **REF C** | **ALT T** | **REF -** | **ALT G** |
| D00201.001 | CNOT4 | **0.238** | **7.301** | -0.167 | -0.191 | 0.026 | -0.080 | 4.868 | 4.868 | **4.751** | **2.693** | -0.101 | -0.143 |
| D00061.001 | Foxc2 | -0.286 | -0.366 | 0.071 | 0.060 | **9.498** | **0.340** | **1.874** | **6.322** | 1.255 | 1.143 | 0.184 | 0.178 |
| D00062.001 | Foxg1 | -0.459 | -0.140 | 0.328 | 0.449 | **12.364** | **0.273** | **1.100** | **7.664** | 0.495 | 0.476 | -0.654 | -0.526 |
| D00004.001 | Foxj2 | -0.457 | -0.439 | -0.283 | -0.286 | **9.560** | **-0.352** | -0.072 | 0.585 | 0.669 | 0.390 | -0.539 | -0.486 |
| D00005.001 | Foxo1 | -0.166 | -0.166 | 0.232 | 0.000 | **6.819** | **-0.091** | 0.828 | 1.424 | 0.191 | 0.172 | -0.161 | -0.162 |
| D00008.001 | Foxp1 | -0.083 | -0.083 | 0.028 | 0.031 | **7.248** | **0.107** | 0.458 | 0.698 | 0.050 | 0.072 | 0.051 | 0.051 |
| D00009.001 | Foxp2 | -0.085 | -0.076 | -0.021 | -0.021 | **10.152** | **0.153** | 0.238 | 0.343 | 0.092 | 0.033 | -0.085 | -0.085 |
| D00011.001 | Irf2 | 0.014 | -0.011 | 0.748 | 0.756 | 0.118 | -0.101 | 4.118 | 4.029 | **0.876** | **10.185** | 0.176 | 0.062 |
| D00619.003 | RFX5 | -0.785 | -0.312 | 3.475 | 1.148 | -1.674 | -1.714 | 2.523 | 2.181 | 1.659 | 1.780 | **5.132** | **-1.959** |
| D00072.001 | Rfx7 | 0.107 | 0.113 | **11.192** | **3.280** | -0.198 | -0.241 | -0.174 | -0.215 | 0.077 | 0.067 | **30.895** | **0.021** |

## Supplementary Figure S4: RFX7, RFX5, and RFX3 vs THEMIS expression in double negative (Q), double positive (Q), and αβ entry T cells, the cell types with the highest RFX7/5/3 expression in thymus in single cell RNA sequencing of developing human immune system (A,B,C).

Light dots include all thymous cell types expressing *THEMIS*, as listed in Supplementary Figure S1. Data from *Suo et al, Science 2022*, accessed through <https://developmentcellatlas.cellgeni.sanger.ac.uk/fetal-immune/pfi/>.


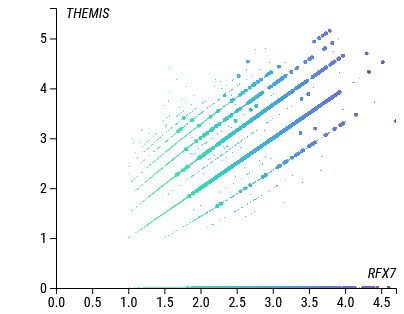

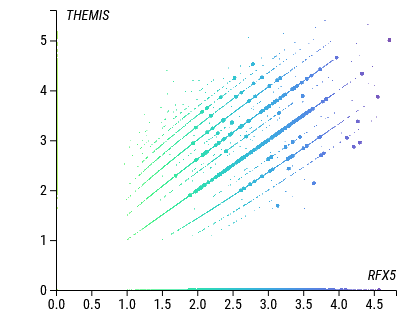

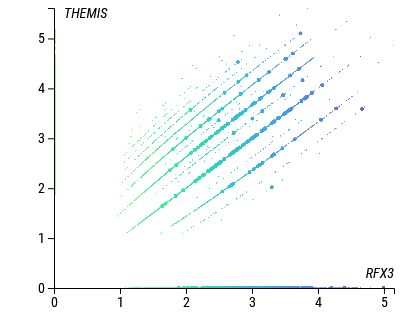


## Supplementary Figure S5: Transcription factor (TF) binding affinity for the 58 SNPs on the chromosome 5p22.33 region, calculated with sTRAP software for all 1241 TFs with JASPAR matrices.

A: -log10(p-value) for REF and ALT alleles. Variants with difference in –log10(p) >2 are indicated with light red; the three top TFs for rs138300818 (Rfx4, Rfxdc2, Rfx3) are indicated with red. B: Histogram of difference in –log10(p REF) vs –log10(p ALT). Red lines indicate difference in P-value for rs138300818 REF and ALT alleles for Rfx4, Rfxdc2, and Rfx3 (from right to left).


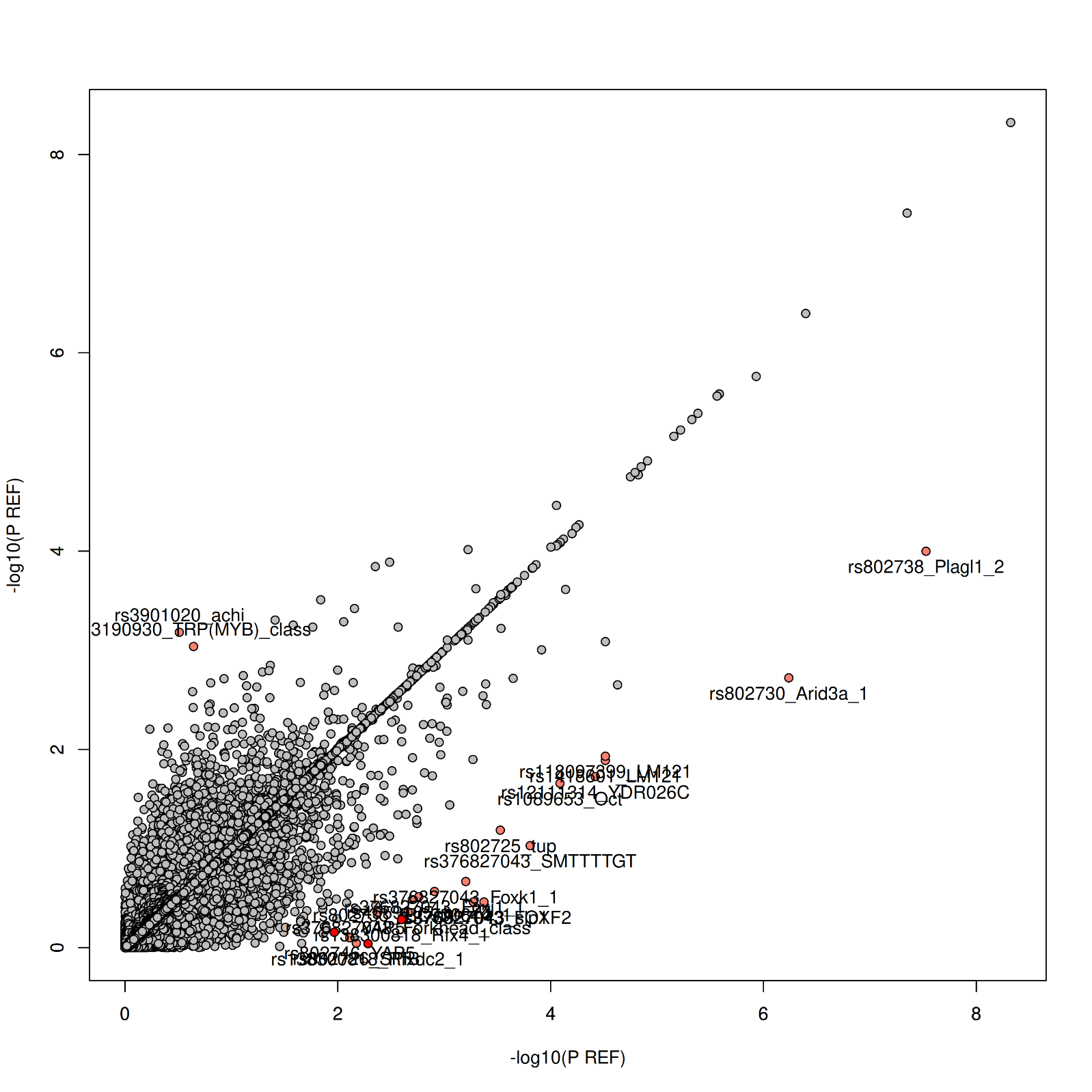


**A**

**B**


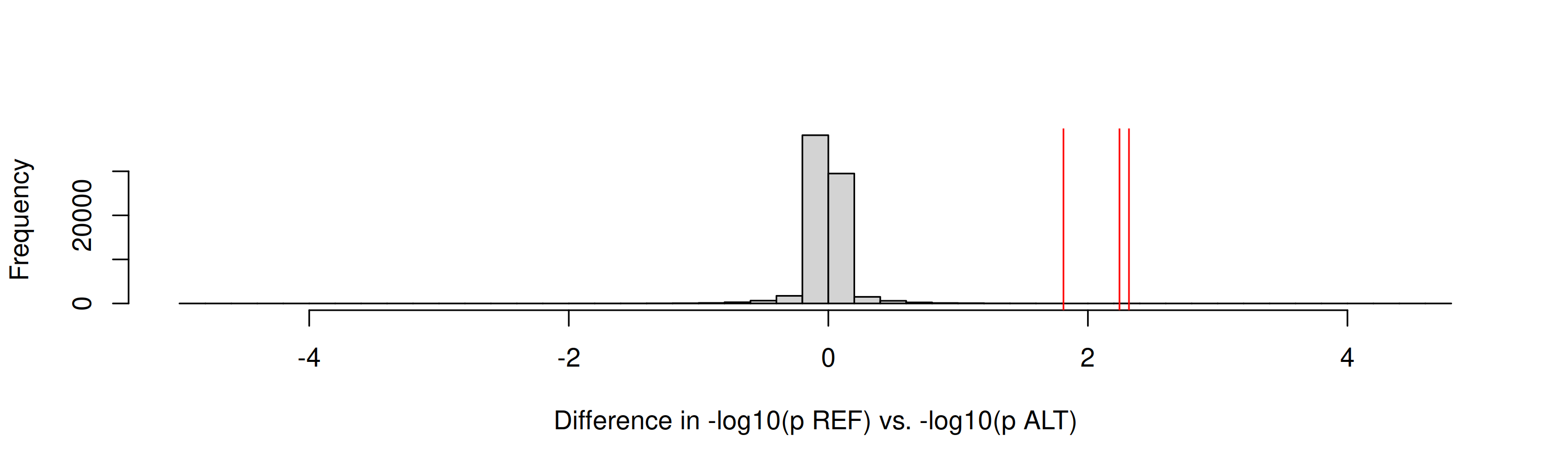


## Supplementary **Figure S6. Non-coding RNA transcription overlapping** rs142852921 (SKAP2)**.**

**
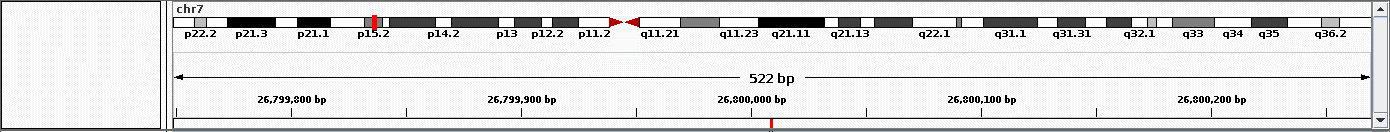
**

**
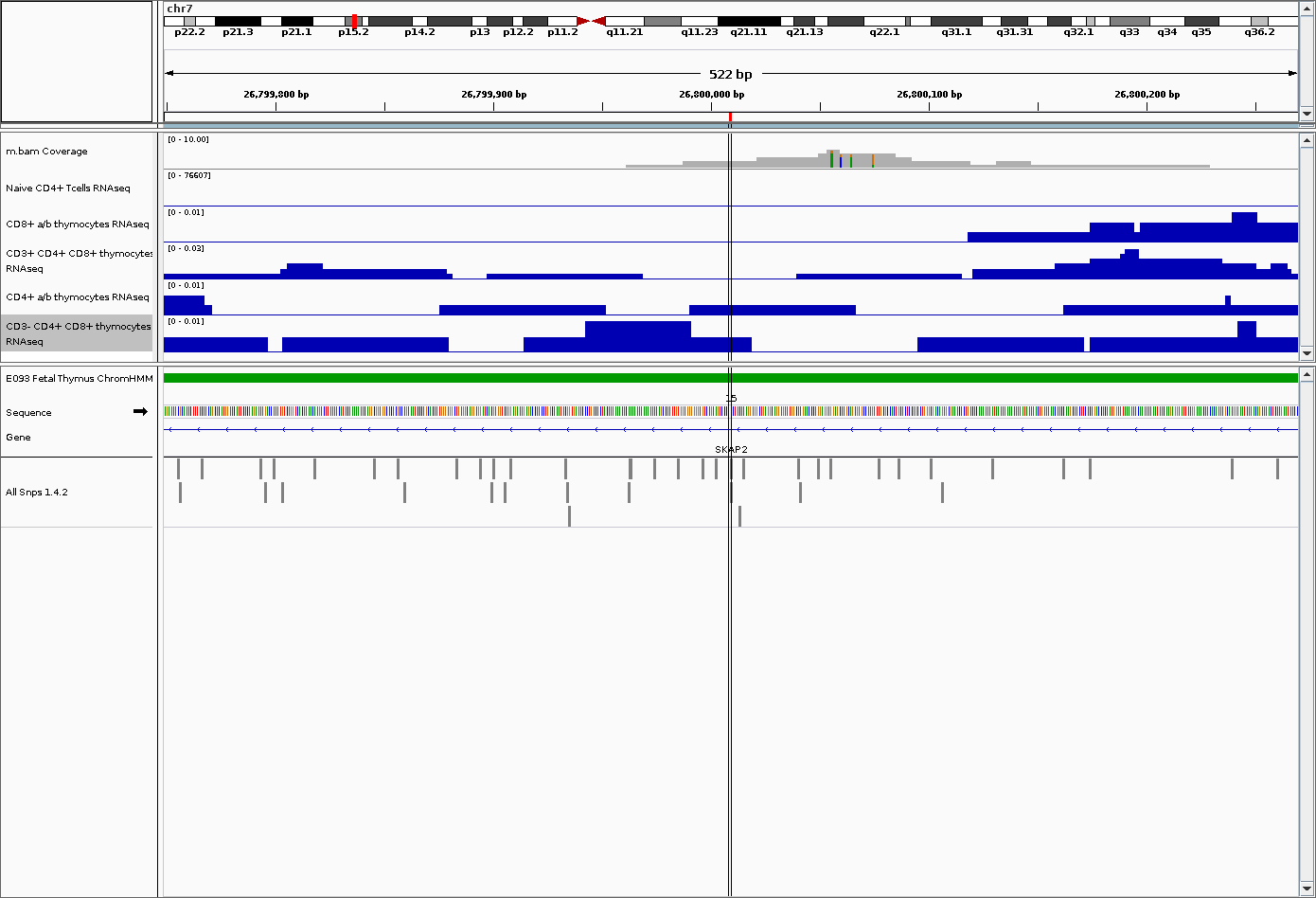
**

## Supplementary Figure S7. Flow chart describing the analysis process for identifying thymocyte histone modification motifs and their integration with age-at-diabetes or T1D SNPs.


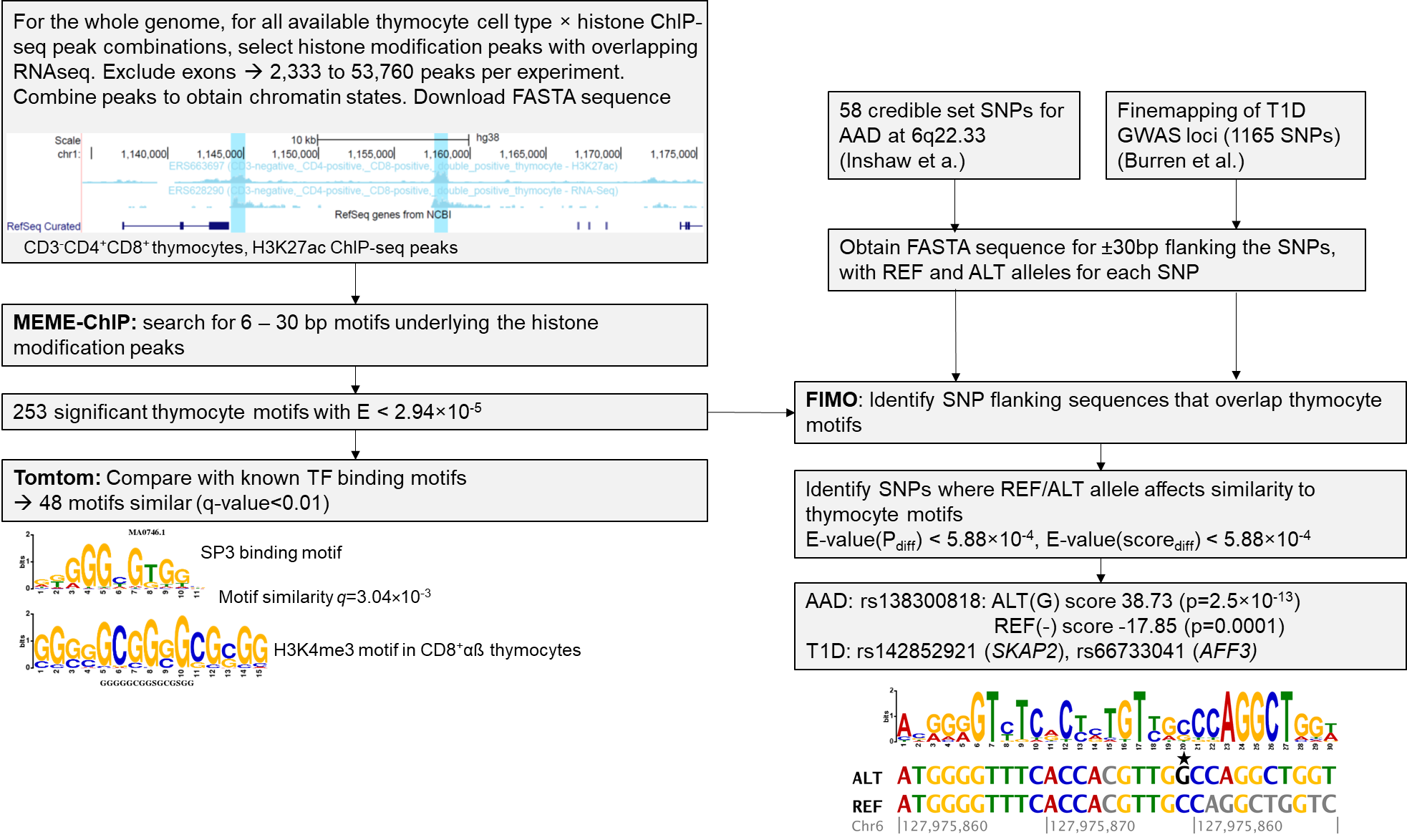

Supplement: Supplementary file 1 — Supplementary Information. [file 41598_2022_18296_MOESM1_ESM.docx]
